# Epilithic diatom communities of selected streams from the Lerma-Chapala Basin, Central Mexico, with the description of two new species

**DOI:** 10.3897/phytokeys.88.14612

**Published:** 2017-10-11

**Authors:** Demetrio Mora, Javier Carmona, Regine Jahn, Jonas Zimmermann, Nélida Abarca

**Affiliations:** 1 Botanischer Garten und Botanisches Museum Berlin–Dahlem, Freie Universität Berlin, Königin–Luise–Straße 6–8, 14195 Berlin, Germany; 2 Facultad de Ciencias, Universidad Nacional Autónoma de México, Circuito Exterior s/n, Ciudad Universitaria, Coyoacán, 04510 México, D.F., México

**Keywords:** Central Mexico, diatom communities, epilithon, indicator species, Lerma-Chapala Basin, mountain streams, new species

## Abstract

The Lerma-Chapala Basin, in Central Mexico, is geologically heterogeneous, climatically diverse and boasts high biodiversity, lying within two Biodiversity Hotspots, namely Mesoamerica and the Madrean Pine–Oak Woodlands. Epilithon and water samples were collected in the basin from 14 sampling sites three times each, two sampling campaigns during the rainy season and one in the dry season. A total of 274 infrageneric taxa in 48 genera were recorded. The taxonomic composition observed was dominated by taxa from the genera *Nitzschia*, *Gomphonema*, *Pinnularia*, *Navicula*, *Sellaphora* and *Eunotia*. About a third of the taxa found could not be identified to the species level. From those unidentified morphodemes, two are described as new species, namely *Brachysira altepetlensis* and *Sellaphora queretana*. Furthermore, *Eolimna rhombica* is transferred to *Sellaphora*. Canonical Correspondence Analysis (CCA) revealed that specific conductivity and pH were the main environmental factors driving the community composition observed. Three groups of samples were identified after the CCA: 1) characterized by acidic waters and low conductivity; 2) with circumneutral waters, low specific conductivity and high temperature and phosphorous concentrations; and 3) characterized by circumneutral waters, high conductivity and low nitrogen concentrations. The indicator value method (IndVal), based on the relative abundance and relative frequency of the most abundant taxa was calculated based on the groups observed in the CCA, identifying the characteristic taxa for each of the three groups.

## Introduction

Lotic environments, i.e. streams, are unidirectional flows of water. They are characterized by a broad spatial (i.e. substrate, slope, vegetation) and temporal (i.e. water velocity, light) heterogeneity, which determines the specialized biota that inhabit them (Giller and Malmqvist 1998, Allan and Castillo 2007). Stream diatoms have features that allow them to thrive in flowing waters, such as the morphological and physiological ability to adhere directly or by means of stalks or mucilage pads to different substrate types to avoid being dragged away by water. Apart from water velocity, physical and chemical variables of the water such as temperature, pH, specific conductivity and nutrient concentrations are determining factors for diatom composition and community structure (Bellinger and Sigee 2010, Stevenson et al. 2010).

Even though there is mounting evidence of the applied use of diatoms as indicators of environmental change in lotic environments (Kelly 1998, Potapova and Charles 2002, 2007, Smol and Stoermer 2010), diatom studies from Mexican streams are relatively scarce, despite the increasing pressure these environments are facing to satisfy human demand for clean water.

Diatom studies of lotic environments from Mexico have been mostly focused on the center of the country: Antigua River Basin (Vázquez et al. 2011); Balsas River Basin (Valadez-Cruz et al. 1996, Bojorge-García et al. 2010, 2014), Lerma-Chapala Basin (Abarca-Mejía 2010, Segura-García et al. 2010, 2012, 2016, Mora et al. 2015), Mexico Basin (Ramírez-Vázquez et al. 2001, Ramírez-Vázquez and Cantoral-Uriza 2003, Bojorge-García and Cantoral-Uriza 2007, Carmona-Jiménez et al. 2016); Pánuco River Basin (Cantoral-Uriza et al. 1997) and Papaloapan River Basin (Tavera et al. 1994). Most of these studies focused on the flora *per se* but also on community structure and bioindication. Despite the research done, the diatom diversity of the region seems to be low due to clustering of taxa into species complexes and force–fitting into already described taxa.

The studies conducted in the Lerma-Chapala Basin have been focused on the polluted Lerma River and some of its main tributaries (Abarca-Mejía 2010, Segura–García et al. 2010, 2012, 2016, Mora et al. 2015). But no study has been conducted so far on the headwater streams of the basin, which are important in the establishment of reference conditions for biological integrity evaluations based on regional characteristics of the streams and its associated diatom flora (Stoddard et al. 2006, Tornés et al. 2007).

In order to contribute to the studies done in the Lerma-Chapala Basin, one of the most important basins of the country regarding population and trade, the aims of this study are: to document the epilithic diatom diversity from selected headwater and midland streams from the Lerma-Chapala Basin, Central Mexico; to illustrate the most abundant taxa; and to identify the environmental factors that determine the variation observed in diatom composition.

## Methods

**Figure 1.**
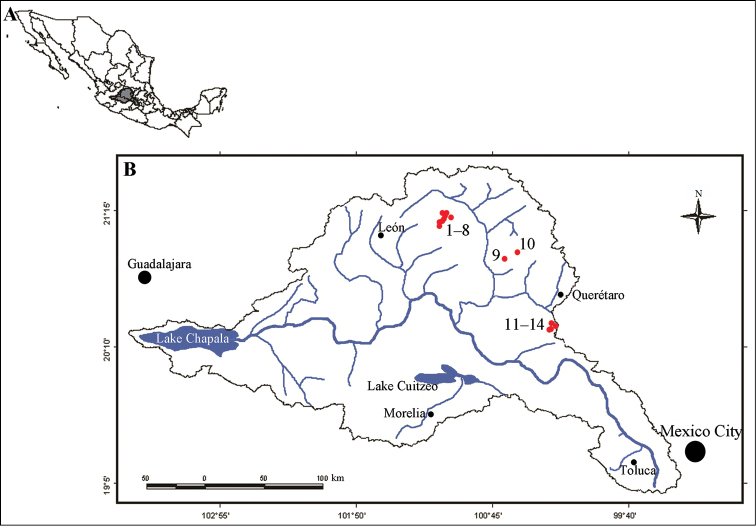
Location of the area of study. A Map of Mexico, showing the location of the Lerma-Chapala Basin in the center of the country. B Location of the 14 sampling sites in the Lerma-Chapala Basin, indicated by red dots. The numbers next to the red dots refer to the name of the sampling site in Table 1.

*Study area.* The Lerma-Chapala Basin is located in Central Mexico, covering an area of 53,591.3 km^2^ (Fig. 1). It is geologically heterogeneous, has a strong elevational gradient, is climatically diverse, has well defined rainy (June to October) and dry seasons (November to May) and boasts high biodiversity. It lies within two Biodiversity Hotspots, namely Mesoamerica and the Madrean Pine–Oak Woodlands (Cotler et al. 2006, CEPF 2017a, b).

This basin is one of the most important centers in the country for agriculture and industry, and has a population of more than 15 million inhabitants (Wester et al. 2005, Cotler et al. 2006). But the Lerma-Chapala Basin is also one of the most environmentally degraded basins in the country, facing serious water related issues because of overexploitation and pollution of surface and underground waters (Aparicio 2001, Wester et al. 2005).

The 14 sampling sites selected for this study are located in the north and central–east sections of the Lerma-Chapala Basin at elevations ranging from 2,000 to 2,400 meters above sea level. Of those 14 sites, one is a perennial spring–fed creek and 13 correspond to streams that have water during most part of the year (Fig. 1, Table 1). Sampling sites 1–8 are located at the foothills of the Sierra de Santa Rosa, an oak–forested mountain range of priority for the conservation of biodiversity in Mexico (Arriaga et al. 2000); the mean temperature of the area is 16.1 °C and the average rainfall is 642 mm (CNA 2017a). Sites 9 and 10 are in a flat area dominated by shrubland and subsistence agriculture; the mean air temperature is 18.8 °C and the average rainfall is 566 mm (CNA 2017b). Sites 11–14 are located at the foothills of a small mountainous area dominated by pine–oak forests and subsistence agriculture; the mean air temperature is 15.6 °C and the average rainfall is 774 mm (CNA 2017c).

**Table 1.** Sampling sites in the Lerma-Chapala Basin, including site number and name, type of water body, geographical coordinates and elevation.

Site		Water body	Latitude (N) / Longitude (W)		Elevation (m a.s.l)
1	La Mesa	Stream	21°05'28.69"N, 101°08'18.98"W	2215
2	Calvillo	Stream	21°06'50.40"N, 101°08'04.10"W	2138
3	Ojo de Agua de Calvillo	Stream	21°07'41.80"N, 101°07'04.50"W	2102
4	Peña Colorada	Stream	21°09'03.84"N, 101°05'58.96"W	2110
5	San Martín	Stream	21°09'24.50"N, 101°03'11.30"W	2017
6	Paredones	Stream	21°11'20.60"N, 101°06'53.40"W	2089
7	La Laborcilla 1	Stream	21°11'04.70"N, 101°06'14.60"W	2076
8	La Laborcilla 2	Stream	21°11'20.10"N, 101°05'37.90"W	2065
9	El Membrillo	Stream	20°50'21.22"N, 100°38'43.46"W	2114
10	Guanajuatito	Spring fed-creek	20°53'23.98"N, 100°32'30.72"W	2120
11	Los Ailes 1	Stream	20°19'58.72"N, 100°15'17.09"W	2358
12	Laguna de Servín 1	Stream	20°18'18.10"N, 100°17'38.10"W	2409
13	Laguna de Servín 2	Stream	20°18'45.20"N, 100°17'25.60"W	2409
14	Los Ailes 2	Stream	20°20'50.20"N, 100°16'45.50"W	2317

*Sampling.* Water and epilithon samples were collected three times from each sampling site in: September/October 2013, rainy season (sampling campaign I); February 2014, dry season (sampling campaign II); and September 2014, rainy season (sampling campaign III); resulting in 42 water and epilithon samples. Each epilithon sample was collected from five cobbles across a transversal section of the stream, brushing with a disposable toothbrush ten square centimeters of epilithic growth from each of the five cobbles to make a composite sample, fixed in 70% alcohol. *In situ* measurements of pH, water temperature, specific conductivity and total dissolved solids were recorded using a Hanna multi–sensor (HI 991300, California, USA). Dissolved oxygen was recorded with an YSI–85 oxygen meter (YSI, Ohio, USA). Dissolved oxygen saturation percentages were calculated from dissolved oxygen data according to correcting factors for elevation and water temperature. Specific conductivity values were corrected to 25°C. Water velocity was recorded with a Global Water FP111 velocity meter (Texas, USA). At each sampling site, a 500 ml sample of water was filtered through 0.22 μm and 0.45 μm filter membranes (Millipore, Massachusetts, USA) and collected in sterile polypropylene bottles for chemical analysis. Samples were kept cold and in the dark before laboratory analysis. The subsequent chemical laboratory analyses were adapted from Standard Methods for the Examination of Water and Wastewater and analyzed using a DR 3900 laboratory Spectrophotometer (Hach Company, Loveland, Colorado) (HACH 2003, APHA 2005): nitrite nitrogen (NO_2_––N), nitrate nitrogen (NO_3_––N), ammonium nitrogen (NH_4_^+^–N), soluble reactive phosphorus (SRP, in theory, mostly in the form of orthophosphate, PO_4_^3–^–P) and total alkalinity (as CaCO_3_). Dissolved inorganic nitrogen (DIN) was calculated as the sum of the three inorganic nitrogen forms in water (nitrites, nitrates and ammonium).

The Riparian Forest Quality index (QBR from its Catalan abbreviation) was calculated in order to evaluate the riparian habitat quality (Munné et al. 2003). This index evaluates quantitatively four components of the riparian habitat: 1) Total riparian vegetation cover, evaluates the vegetation cover of all plants except for annuals and also taking into account the connectivity between the riparian area and surrounding terrestrial vegetation. 2) Vegetation cover structure, it assesses the structural complexity of the riparian habitat, which is determined by the percent coverage and patch distribution of trees, shrubs and aquatic plants. 3) Cover quality, takes into account the number of native tree and shrub species (dependent of the river type) and also evaluates if the river has alterations such as man-made structures, presence of alien species and garbage. To determine the river type, the following geomorphological criteria are evaluated: slope and form of the riparian zone, presence of islands in the river and percentage of hard substrata. 4) River channel alterations, evaluates how pristine or altered is the river, considering if the river has been permanently channelized, if there are rigid structures or fluvial terraces constraining the flow. Each component of the index scores between 0 and 25, therefore the index score go from 0 to 100. The index has five classes: natural condition, good quality, fair quality, poor quality and bad quality. The native vegetation, needed to calculate this index, was identified following Zamudio et al. (1992), Carranza-González (1995), Carranza-González and Madrigal-Sánchez (1995), Calderón de Rzedowski and Rzedowski (2001), Rzedowski and Calderón de Rzedowski (2004).

*Diatom analysis.* Fractions of the diatom samples were cleaned by adding aliquots of 35% hydrogen peroxide and heating at 80°C until no bubbling was observed. After the digestion was completed, peroxide remnants were removed by rinsing at least three times with distilled water. Samples were finally diluted with distilled water in order to avoid high concentrations of valves and sediment. Three permanent slides per sample were made using the high refraction index mounting medium Naphrax^®^. The slides were scanned and the diatoms photographed under the light microscope (LM) in order to account for diatom diversity, using a Zeiss Axioscope microscope with Differential Interference Contrast equipped with an AXIOAM MRc camera. In order to estimate the relative abundance of the taxa, a minimum of 500 valves per sample were counted and identified with the 100x immersion oil objective. Aliquots of cleaned sample material for scanning electron microscopy observations were mounted on stubs, sputter-coated with gold-palladium and observed under a Hitachi FE 8010 scanning electron microscope (SEM) operated at 1.0 kV. Samples and slides are stored at the Diatom Collection of the Botanical Garden and Botanical Museum Berlin–Dahlem, Freie Universität Berlin. Diatoms were identified to the lowest taxonomical level possible using monographs as well as papers for particular taxa (Suppl. material 1). Taxa identified with ‘cf.’ (*confer*) before the epithet indicate that it could be that taxon but the taxonomic identity is still uncertain, ‘aff.’ (*affinis*) that it has some similarity to the taxon but it is not conspecific and ‘sp.’ (species) was used when the taxon showed no similarity with any known species after the literature review.

*Data analysis.* Only taxa with relative abundance ≥1% were included in the statistical analyses, resulting in 105 diatom taxa. Diatom abundances were transformed using Hellinger’s transformation, which is suited to large abundance datasets with lots of low counts and zeros (Legendre and Gallagher 2001).

From the initial dataset composed of 42 samples, only 39 were used for the analysis of running waters, i.e. those streams with water velocity records in at least one of the sampling campaigns; the three samples of site 10 were omitted since no water velocity was recorded in this spring-fed creek at any of the three sampling campaigns, with 10 cm/s being the detection limit of the water velocity meter. All the environmental variables, except for temperature, pH and water velocity were transformed using log_10_ (x+1) because they had skew distributions. Distribution tests were run in Statistica 8.0.

Multivariate analyses were performed to explore gradients in diatom composition and its relation to environmental factors. Detrended Correspondence Analysis (DCA) was used to estimate gradient lengths. The first four axes showed lengths of 5.7, 3, 2.3 and 2.2, suggesting a strong unimodal response, meaning that a method based on unimodal models like Canonical Correspondence Analysis (CCA) would be appropriate for subsequent ordination. CCA was run to identify variation in species composition that can be determined by environmental variables. Since not all the environmental variables influence diatom distributions independently, CCA with forward selection and unrestricted Montecarlo permutation tests was used (999 permutations, *p*<0.05). All ordinations were done using CANOCO 4.5 for Windows (ter Braak and Ṧmilauer 2002), with downweighting of rare species in all cases.

The indicator value method (IndVal) (Dufrêne and Legendre 1997) was used to identify the most characteristic species of the groups visualized after the CCA. This method combines the specificity (relative abundance) and fidelity (relative frequency) of a species to a given group. The indicator value of a species is given in percentage, reaching its maximum when all the individuals of a species are present at all the sites of a single group. Species with high indicator values >50% are considered to be good indicators; species with values between 25–50% might be regarded as detector species of change, therefore detector species can be present in more than one group (Tornés et al. 2007, Carmona-Jiménez et al. 2016). IndVal calculations were run in PC–ORD 4 (McCune and Mefford 1999) with untransformed abundance data. The statistical significance of the IndVal was tested with a randomization Montecarlo test (10,000 permutations, *p*<0.05). The Shannon-Wiener diversity index and Pielou evenness index were calculated as in Peet (1974) for the groups visualized after the CCA.

## Results

*Species composition and taxonomy.* A total of 196 taxa (species and varieties) were found while performing the counts to determine relative abundances. Seventy-eight additional taxa were observed by scanning the whole slides looking for rare taxa, bringing the total diversity to 274 taxa belonging to 48 genera (Suppl. material 1). Sixty-three taxa are new records for the Lerma-Chapala Basin. The most common taxa (relative abundances ≥1% in at least one sample), illustrated here (Figs 2–117), were included in subsequent statistical analyses.

**Figures 2–34.**
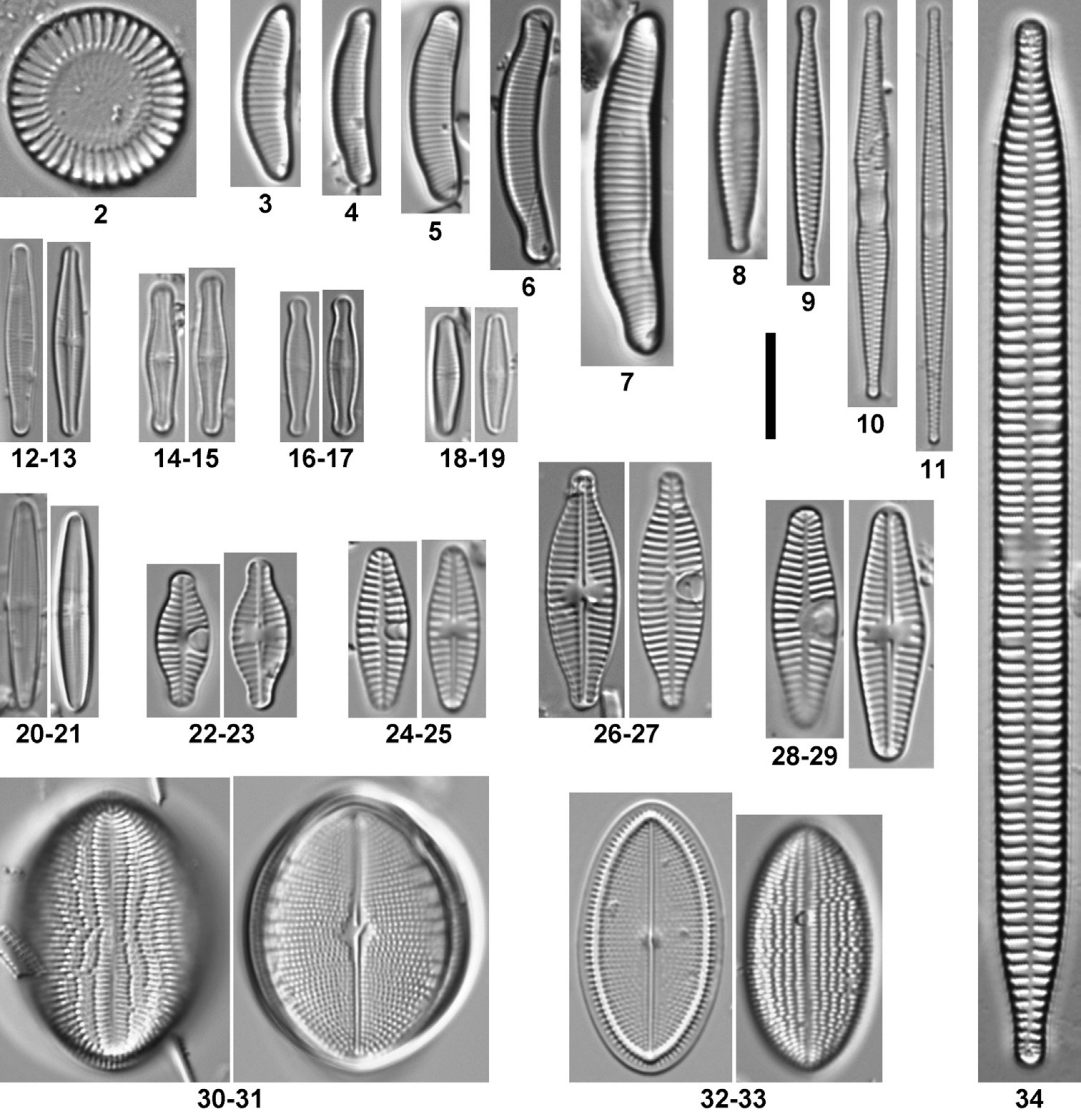
Overview of the most abundant taxa (≥ 1% relative abundance in at least one sample). **2** *Cyclotella meneghiniana* **3** Eunotia cf. meridiana **4** *Eunotia* sp. 1 **5** *Eunotia* sp. 3 **6** *Eunotia* sp. 2 **7** *Eunotia minor* **8** *Fragilaria pectinalis* **9** *Fragilaria austriaca* **10** *Fragilaria bidens* **11** *Fragilaria tenera* **12–13** *Achnanthidium* sp. 5 **14–15** Achnanthidium aff. catenatum **16–17** *Achnanthidium* sp. 1 **18–19** *Achnanthidium minutissimum* **20–21** *Achnanthidium* sp. 4 **22–23** *Planothidium rostratum* **24–25** *Planothidium victori* **26–27** *Planothidium incuriatum* **28–29** *Planothidium cryptolanceolatum* **30–31** *Cocconeis pediculus* **32–33** *Cocconeis* sp. 2 **34** *Ulnaria ulna*. Scale bar 10 μm.

**Figures 35–77.**
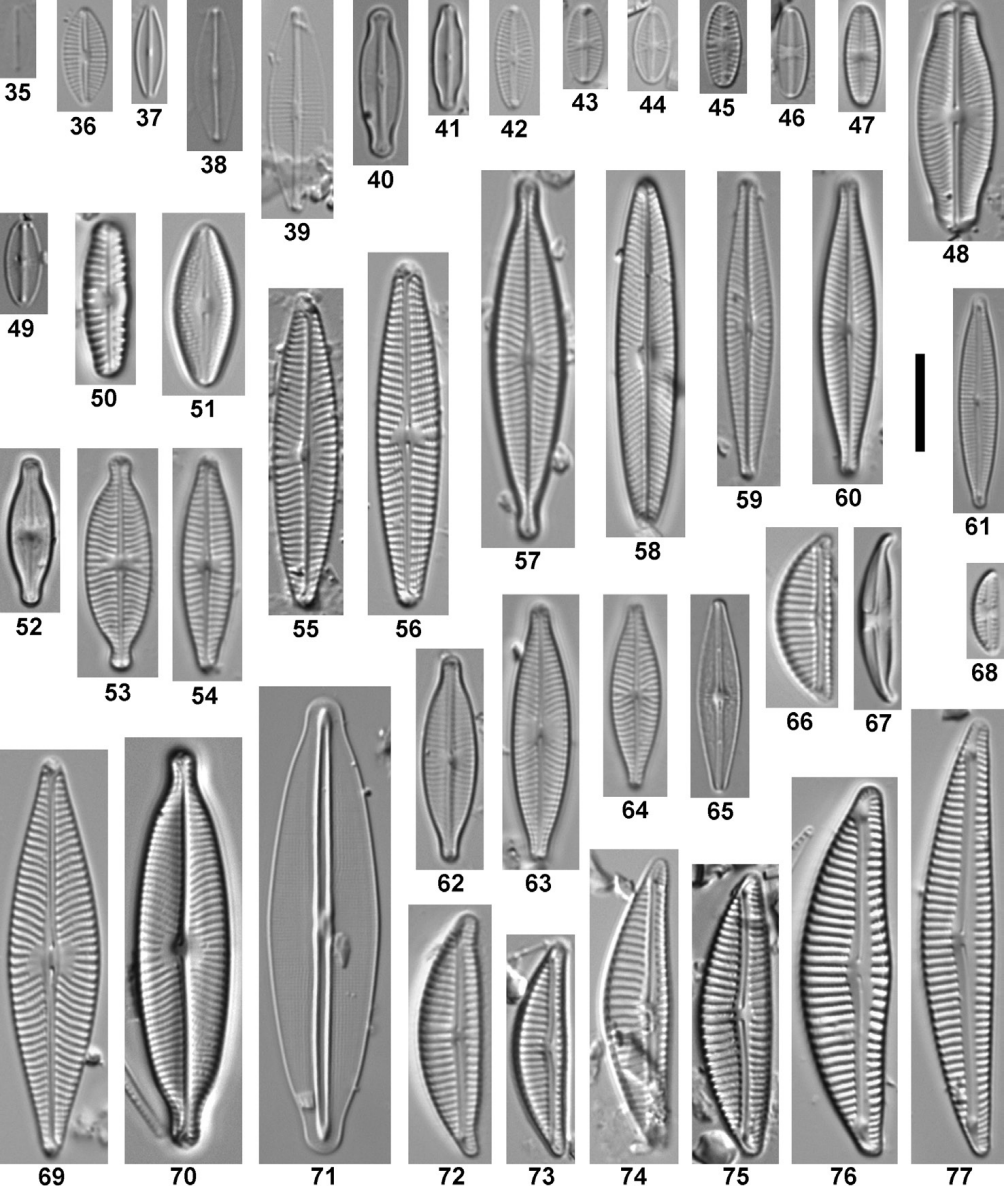
Overview of the most abundant taxa (≥ 1% relative abundance in at least one sample). **35** *Fistulifera saprophila* **36** *Craticula subminuscula* **37** *Craticula* sp. 2 **38** *Craticula molestiformis* **39** Craticula cf. pumilio **40** *Sellaphora cosmopolitana* **41** *Sellaphora* sp. 3 **42** *Eolimna* sp. 1 **43** *Sellaphora nigri* **44** *Sellaphora madida* **45** *Sellaphora queretana* **46** *Sellaphora atomoides* **47** *Sellaphora saugerresii* **48** *Sellaphora pupula* **49** *Mayamaea permitis* **50** *Reimeria sinuata* **51** *Diadesmis confervacea* **52** *Nupela wellneri* **53** *Geissleria decussis* **54** *Navicula veneta* **55** *Navicula erifuga* **56** *Navicula libonensis* **57** *Navicula capitatoradiata* **58** *Navicula symmetrica* **59** *Navicula notha* **60** Navicula cf. cryptocephala **61** Encyonopsis cf. thienemannii **62** *Navicula gregaria* **63** *Navicula cryptocephala* **64** *Navicula reichardtiana* **65** *Brachysira altepetlensis* **66** *Encyonema minutum* **67** *Halamphora montana* **68** *Amphora pediculus* **69** *Navicula trivialis* **70** *Navicula rostellata* **71** *Frustulia crassinervia* **72** *Encyonema brevicapitatum* **73** *Encyonema minutiforme* **74** Encyonema cf. minutiforme **75** Encyonema cf. hebridiforme **76** *Encyonema jemtlandicum* **77** *Encyonema pergracile*. Scale bar 10 μm.

**Figures 78–117.**
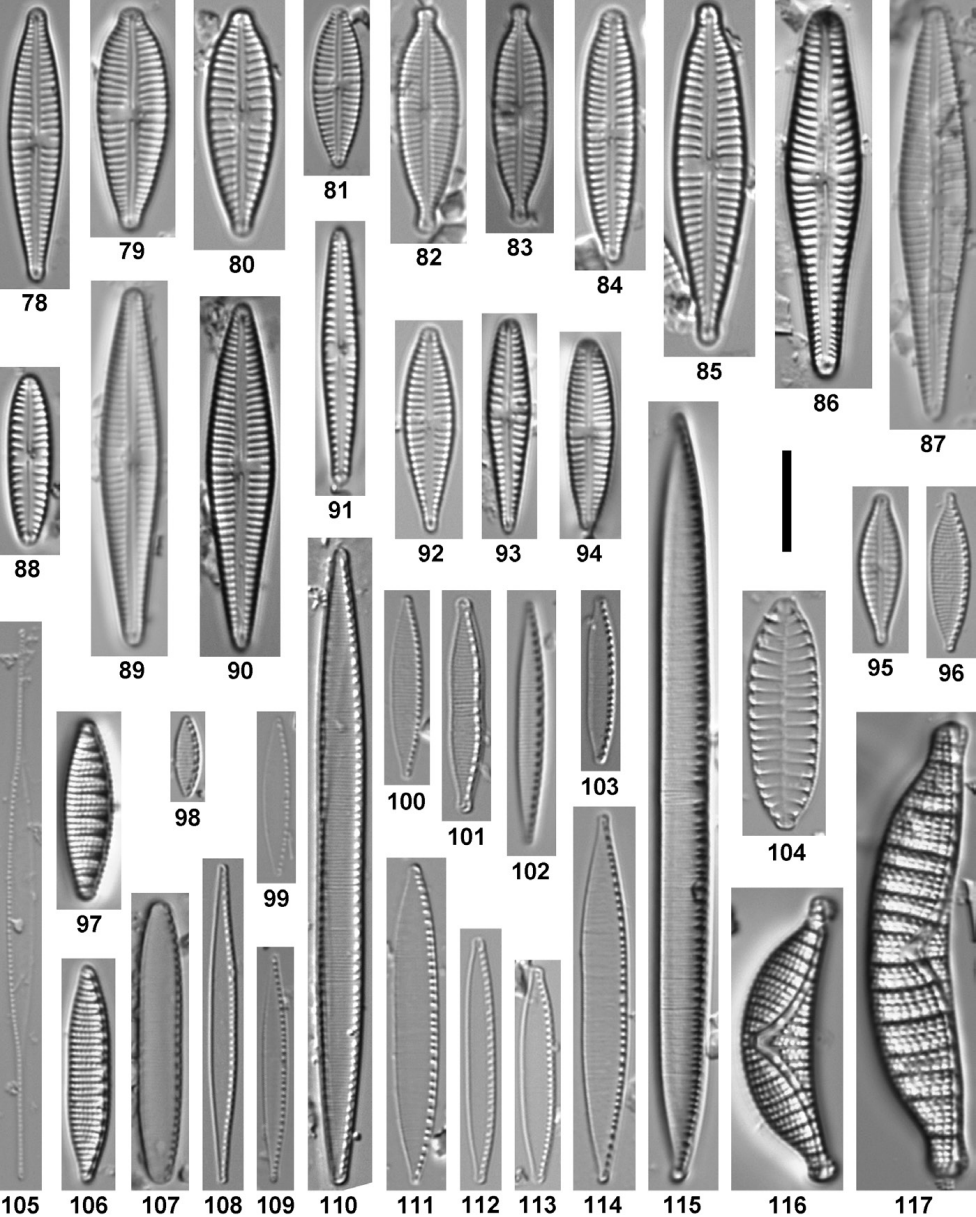
Overview of the most abundant taxa (≥ 1% relative abundance in at least one sample). **78** *Gomphonema exilissimum* **79** *Gomphonema parvuliforme* **80** Gomphonema cf. parvuliforme **81** *Gomphonema parvulum* **82** *Gomphonema lagenula* **83** Gomphonema cf. lagenula **84** Gomphonema aff. sarcophagus **85** Gomphonema aff. mariovense **86** *Gomphonema subclavatum* **87** *Gomphonema stonei* **88** *Gomphonema pumilum* **89** *Gomphonema graciledictum* **90** *Gomphonema naviculoides* **91** *Gomphonema minusculum* **92** *Gomphonema* sp. 4 **93** *Gomphonema* sp. 2 **94** *Gomphonema innocens* **95** Gomphonema aff. parvulius **96** *Nitzschia desertorum* **97** *Nitzschia semirobusta* **98** *Nitzschia inconspicua* **99** *Nitzschia* sp. 1 **100** *Nitzschia supralitorea* **101** Nitzschia cf. hantzschiana **102** *Nitzschia fonticola* **103** *Nitzschia perminuta* **104** *Surirella angusta* **105** *Nitzschia acicularis* **106** *Nitzschia amphibia* **107** *Nitzschia communis* **108** *Nitzschia gracilis* **109** *Nitzschia paleacea* **110** *Nitzschia intermedia* **111** *Nitzschia palea* **112** Nitzschia palea var. tenuirostris **113** Nitzschia palea var. debilis **114** *Nitzschia balcanica* **115** *Nitzschia linearis* **116** *Epithemia sorex* **117** *Epithemia adnata*. Scale bar 10 μm.

A high specific taxa richness was found among the genera *Nitzschia* (35 taxa), *Gomphonema* (26 taxa), *Pinnularia* (21 taxa), *Navicula* (19 taxa), *Sellaphora* (18 taxa) and *Eunotia* (16 taxa). About a third of the diversity found, 94 taxa, did not fit completely into already described species. Most of the taxa were found in relatively low abundances while further scanning the slides under the LM after the enumeration of 500 valves; when scanning samples under the SEM, some of those rare unidentified taxa were found but in several cases not. When the taxa were found under the SEM, not enough valves were observed for reliable identification. This is why only two new species from those 94 unidentified taxa are here described as new, one belonging to the genus *Brachysira* and the other to *Sellaphora*. Furthermore, one *Eolimna* species is transferred to *Sellaphora*, this species sharing the same morphology of areolae as the *Sellaphora* species here described as new.

### Brachysira altepetlensis

Taxon classificationPlantaeNaviculalesBrachysiraceae

D.Mora, R.Jahn & N.Abarca sp. nov.

urn:lsid:ipni.org:names:

Figs 118–132

#### Holotype.

B 40 0042006; Figure 121 represents the holotype.

#### Isotypes.

B 40 0042007 (SEM stub), QMEX DIAT0001 (Slide).

Cleaned unmounted material is available under the numbers B 40 0042008 and QMEX DIAT0002.

#### Type locality.

Paredones stream, on the outskirts of Paredones village, Dolores Hidalgo, Guanajuato, Mexico (21°11'20.60"N; 101°06'53.40"W; 2089 m a.s.l). Collected by Demetrio Mora on 07.09.2014.

#### Registration.

http://phycobank.org/100101

#### Description.

**Figures 118–132.**
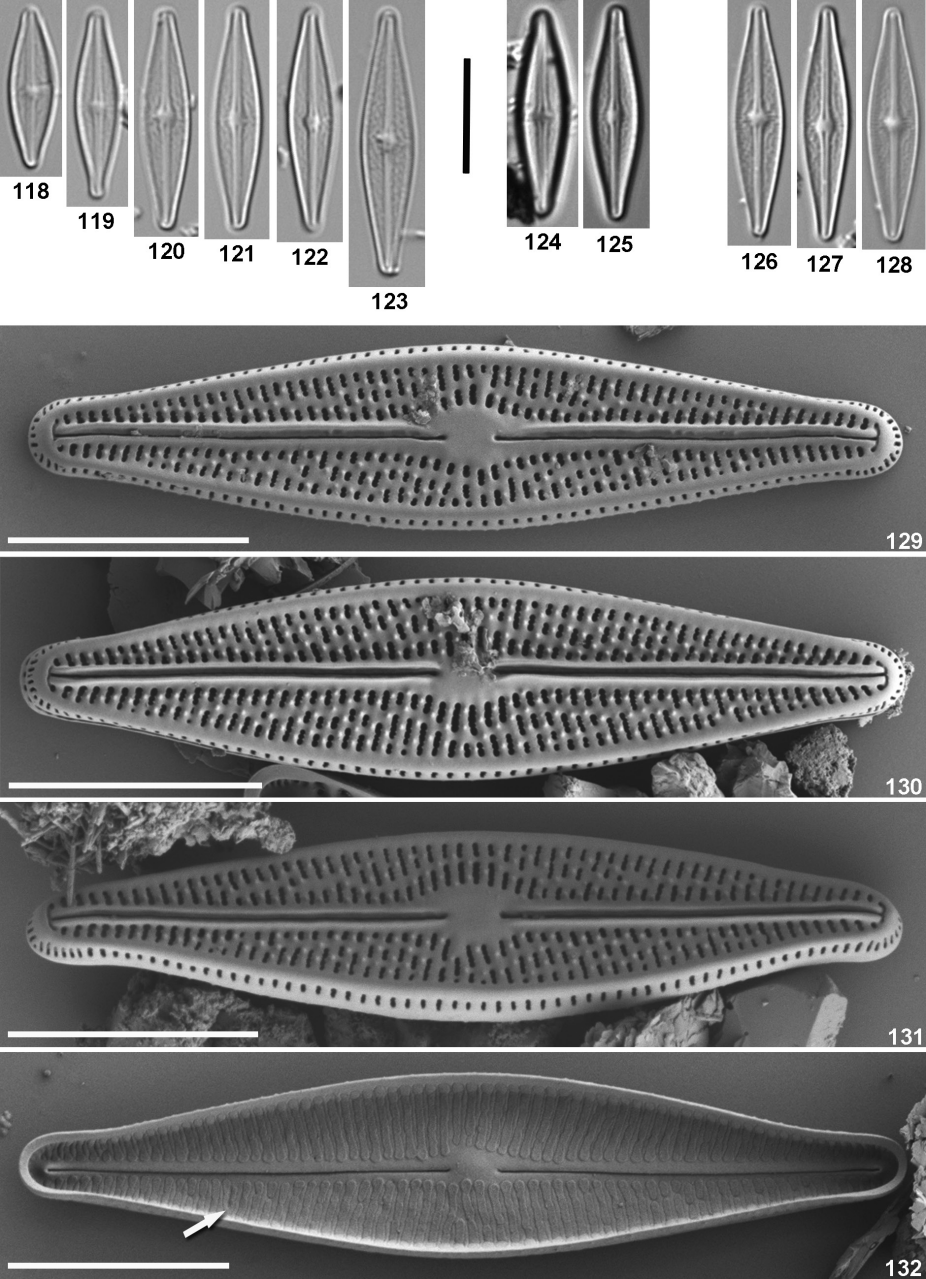
*Brachysira altepetlensis* D. Mora, R. Jahn & N. Abarca, sp. nov. LM (**118–128**) and SEM (**129–132**). **118–123** type material, from Paredones stream, Guanajuato, Mexico, collected on 07.09.2014 **121** designated as holotype **124–125** collected from type location but on 06.10.2013 **126–128** collected from type location but on 09.02.2014 **129–132** from type material: **129–130** external view of entire valves **131** external view of an entire valve showing elongated areolae in the valve mantle **132** internal view of entire valve, showing occlusion of the areolae by hymens. The arrow points at Voigt discontinuity. Scale bars 10 μm (**118–128**); 5 μm (**129–132**).

the valves are lanceolate to linear–lanceolate with rostrate apices. The axial area is narrow–linear throughout the valve and the central area round to elliptical (Figs 118–128). Length: 12.6–23.1 µm, width: 3.2–4.5 µm, length/width ratio: 3.2–5.4; striae in 10 µm: 34–37. The raphe is filiform, slightly sinuous, bordered by a thickened longitudinal siliceous rib on both sides (Figs 129–131). The proximal raphe ends are straight, while the distal raphe endings are T-shaped (Figs 129–131). Internally, the proximal raphe endings are slightly bent to the same side of the valve and distally end in helictoglossa (Fig. 132). The striae are uniseriate and radiate throughout; composed of 2–3 transapically elongated areolae except close to the apices where only one elongated areola is present (Figs 129–131). Striae in the valve mantle are composed of single elongated areola (Fig. 131). In some valves the Voigt discontinuity can be seen (Fig. 132). Internally the areolae are occluded by hymens (Fig. 132). The virgae have irregularly spaced papillae (Figs 129–131).

#### Differential diagnosis.

*Brachysira procera* Lange-Bertalot & Gerd Moser is the species which most closely resembles *B. altepetlensis* in valve outline but is larger (25–60 µm), wider at valve center (4.5–6 µm) and has less striae in 10 µm (27–30) (Lange-Bertalot and Moser 1994). The valve outline of *Brachysira neglectissima* Lange-Bertalot also resembles that of *B. altepetlensis* but the valves of *B. neglectissima* are wider (4.3–4.5 µm), have more striae (36–40), the areolae are arranged in a way that they give the appearance of waves and each single areola is comparatively not as elongated as in *B. altepetlensis* (Lange-Bertalot and Moser 1994). *Brachysira guarrerai* Vouilloud, Sala & Núñez-Avellaneda is also similar in valve outline but the valves are wider (5.5–7 µm), have less striae (26–32) and lack papillae in the interstriae (Vouilloud et al. 2014).

The valve dimensions as well as the striae density of the new species fall within the range of the *Brachysira neoexilis* Lange-Bertalot species complex, but the type population of *B. neoexilis* has clear capitate apices and the larger specimens have a very slightly triundulate valve margins (Lange-Bertalot and Moser 1994). All the other populations from *B. neoexilis* species complex depicted in the original description (Lange-Bertalot and Moser 1994) have subcapitate to capitate apices, not matching at all the outline of *B. altepetlensis*. The specimens depicted in Rumrich et al. (2000), identified as *B. neoexilis* (Pl. 89: figs 18–20), closely resemble *B. altepetlensis* in valve outline but they clearly differ from specimens depicted in the type description of *B. neoexilis* (Lange-Bertalot and Moser 1994). The specimens of *Brachysira* found by Abarca-Mejía (2010) in a spring also in the Lerma-Chapala Basin, closely resemble *B. altepetlensis* in LM, but her identification was based on Rumrich et al. (2000), which led her to identify those valves as *B. neoexilis*.

#### Etymology.

this new *Brachysira* species takes the name from the word “āltepētl” which means “water mountain” in Náhuatl language, that is how the surrounding mountains were used to be named by native people 500 years ago, at the time Spaniards first came to the region.

#### Distribution.

apart from the type locality, this species was also found in four streams sampled for this study, namely Peña Colorada (site 4), San Martín (site 5), La Laborcilla 1 (site 7) and La Laborcilla 2 (site 8), all of these sites were characterized by low specific conductivity (≤ 100 μS/cm) and pH values going from acidic to slightly alcaline (5.1–7.9). But *B. altepetlensis* only reached high relative abundances (>10%) in acidic waters (pH= 5.1–5.8) with low specific conductivity (42–53 μS/cm).

### Sellaphora queretana

Taxon classificationPlantaeNaviculalesSellaphoraceae

D.Mora, N.Abarca & J.Carmona sp. nov.

urn:lsid:ipni.org:names:

Figs 133–144

#### Holotype.

B 40 0042009; Figure 137 represents the holotype.

#### Isotypes.

B 40 0042010 (SEM stub), QMEX DIAT0003 (Slide).

Cleaned unmounted material is available under the numbers B 40 0042011 and QMEX DIAT0004.

#### Type locality.

stream Los Ailes 1, close to the town San Pedro, Huimilpan, Querétaro, Mexico (20°19'58.72"N; 100°15'17.09"W; 2358 m a.s.l). Collected by Demetrio Mora on 18.09.2013.

#### Registration.

http://phycobank.org/100102

#### Description.

**Figures 133–144.**
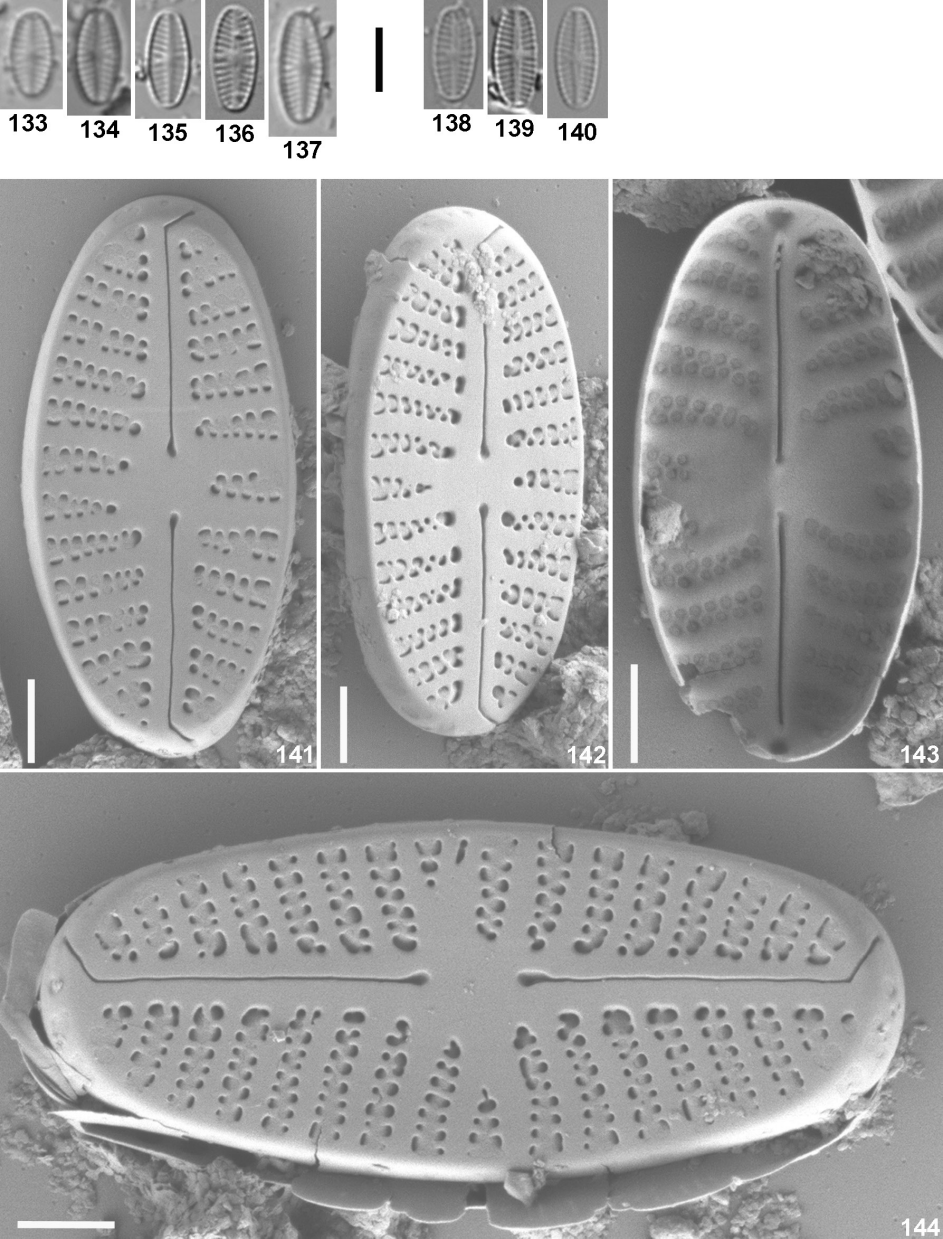
*Sellaphora queretana* D. Mora, N. Abarca & J. Carmona, sp. nov. LM (**133–140**) and SEM (**141–144**). **133–137** type material, from stream Los Ailes 1, Querétaro, Mexico, collected on 18.09.2013 **137** designated as holotype **138–140** population from stream Laguna de Servín 2, collected on 29.09.2013 **141–144** from type material: **141, 142, 144** external views of entire valves **143** internal view of an entire valve. Scale bars 5 μm (**133–140**); 1 μm (**141–144**).

the valves are linear–elliptical with broadly rounded apices (Figs 133–140). The axial area is narrow–linear throughout most of the valve, slightly widening close to the central area. The central area is asymmetrical due to irregular shortenings of the striae bordering it (Figs 141, 142 and 144). Length: 5.6–8.4 µm, width: 2.8–3.9 µm, length/width ratio: 1.9–2.4; striae in 10 µm: 19–22. The raphe is filiform with enlarged proximal raphe endings and slightly deflected to the same side of the valve; the distal raphe endings are strongly bent to the same side of the valve and extended onto the mantle (Figs 141, 142 and 144); the deflection of both proximal and distal raphe endings in external valve face is in the same direction (Figs 141, 142 and 144). Internally, the proximal raphe endings are straight and distally the raphe ends in helictoglossa (Fig. 143). The striae are biseriate and radiate throughout, however becoming uniseriate near the central area (Figs 141, 142 and 144). The areolae are lunate in form and are internally occluded by a hymen (Fig. 143). The hymenes are close to the foramina (seen on external view) (Figs 141, 142 and 144).

#### Differential diagnosis.

there are no known taxa with the same combination of valve outline and areola type. The outline of *S. queretana* resembles that of *Sellaphora chistiakovae* (Kulikovskiy & Lange–Bertalot) C.E. Wetzel, Ector, Van de Vijver, Compère & D.G. Mann; the linear–elliptical forms of *Sellaphora crassulexigua* (E. Reichardt) C.E. Wetzel & Ector; and that of *Sellaphora nigri* (De Notaris) C.E. Wetzel & Ector. But *S. chistiakovae* has uniseriate to irregularly biseriate striae (Kulikovskiy et al. 2010); *S. crassulexigua* and *S. nigri* have uniseriate striae (Wetzel et al. 2015). Taxa with similar striae, with hymenes close to the foramina, include *Sellaphora labernardierei* Beauger, C.E.Wetzel & Ector, *Sellaphora rhombelliptica* (Gerd Moser, Lange–Bert. & Metzeltin) C.E. Wetzel & Ector, *Sellaphora rhombica* (Gerd Moser, Lange–Bert. & Metzeltin) D. Mora, N. Abarca & R. Jahn, comb. nov. (see new combination below) and *Sellaphora thioense* (Gerd Moser, Lange–Bert. & Metzeltin) C.E. Wetzel, Ector, Van de Vijver, Compère & D.G. Mann. But the valves of *S. labernardieri* are linear to linear–elliptical, slightly inflated at the center and have consistently more striae 10 µm (20–28, mainly 24–25) (Beauger et al. 2016). *Sellaphora rhombelliptica* has more striae (25), which are uniseriate and the valves are rhomboelliptic (Moser et al. 1998). *Sellaphora rhombica* has similar number of striae (17–21) but the valve outline is rhombic to rhombic–lanceolate (Moser et al. 1998). *Sellaphora thioense* has slender elliptical valves (2.5–2.8) with higher striae density (27–28) (Moser et al. 1998).

#### Etymology.

this new *Sellaphora* species takes its name from the demonym of the Mexican state Querétaro, from where it was collected.

#### Distribution.

so far only known from the type locality (sampling site 11 in this study) and from stream Laguna de Servín 2 (site 13) located 4 km away from the type location, in acidic waters (pH 5.9–6.2) with low conductivity (77–88 μS/cm).

Based on morphological similarities with other small *Sellaphora* species, *Eolimna rhombica* Gerd Moser, Lange–Bertalot & Metzeltin is transferred to *Sellaphora*:

### Sellaphora rhombica

Taxon classificationPlantaeNaviculalesSellaphoraceae

(Gerd Moser, Lange-Bertalot & Metzeltin) D.Mora, N.Abarca & R.Jahn comb. nov.

urn:lsid:ipni.org:names:

#### Basionym.

*Eolimna rhombica* Gerd Moser, Lange-Bertalot & Metzeltin, 1998, Bibliotheca Diatomologica, vol. 38, p. 156, pl. 23, figs 11–20.

#### Registration.

http://phycobank.org/100103

### Community analysis

The physical and chemical composition of the water from the sampling sites, as well as QBR values are enlisted in Table 2. From the original dataset of 14 environmental variables used in the DCA, total dissolved solids and total alkalinity were highly correlated with specific conductivity and therefore removed from the analysis. Dissolved oxygen and dissolved oxygen saturation percentage were also highly correlated, the latter being removed from further analysis. Dissolved inorganic nitrogen was also removed because it correlated strongly with nitrates. CCA with forward selection and unrestricted Monte Carlo permutations tests (999 permutations, *p*<0.05) identified temperature (*F*=1.60, *p*=0.028), pH (*F*= 2.53, *p*=0.0010), specific conductivity (*F*= 5.07, *p*=0.0010), soluble reactive phosphorous (*F*=1.68, *p*=0.0060) and the Riparian Forest Quality Index (*F*=2.47, *p*=0.0010) as the variables that significantly explained variation in the diatom data. The first two CCA axes accounted for 66.5 % of the cumulative variance of the species – environmental relation, both axes being significant (*p*=0.0010). The first CCA axis was strongly correlated with specific conductivity (inter-set correlation *r*= 0.93) and pH (*r*= 0.80). The second CCA axis was negatively correlated with QBR (*r*= -0.61) and positively correlated with temperature (*r*= 0.44).

**Table 2.** Physical and chemical composition of the water from the sampling sites in the Lerma‒Chapala Basin. Samples were taken in September/October 2013 for sampling campaign I, in February 2014 for the campaign II and in September 2014 for campaign III. T= temperature in °C; Cond= specific conductivity corrected at 25°C (μS/cm); TDS= total dissolved solids as particles per million (ppm); TA= total alkalinity mg/L of CaCO_3_; *v*= water velocity (cm/s); DO= dissolved oxygen (mg/L); DOS= dissolved oxygen saturation percentage; SRP = soluble reactive phosphorous (mg/L); NO_2_^‒^‒N = nitrite nitrogen (mg/L); NO_3_^‒^‒N = nitrate nitrogen (mg/L); NH_4_^+^‒N= ammonium nitrogen (mg/L); DIN= dissolved inorganic nitrogen (mg/L); QBR = Riparian Forest Quality Index.

Sampling campaign	Site	T	pH	Cond	TDS	TA	*v*	DO	DOS	SRP	NO_2_^‒^‒N	NO_3_^‒^‒N	NH_4_^+^‒N	DIN	QBR
I Rainy season	1	14.5	6.7	114	45	30	29	6.5	84	1.09	0.005	0.010	0.005	0.02	85
	2	16.1	7.5	417	173	91	33	8.2	107	0.92	0.005	0.010	0.050	0.06	75
	3	17.8	7.7	422	182	93	39	7.2	98	0.59	0.004	0.015	0.025	0.04	75
	4	26.3	7.4	59	30	12	11	6.5	103	0.59	0.004	0.010	0.020	0.03	55
	5	25.8	7.1	100	51	13	24	6.8	105	0.57	0.003	0.010	0.005	0.02	70
	6	20.0	5.8	48	21	9	15	6.7	94	0.49	0.002	0.010	0.000	0.01	75
	7	23.5	6.1	84	41	23	32	7.2	108	0.68	0.003	0.020	0.000	0.02	50
	8	25.4	6.3	70	35	18	38	5.9	91	0.50	0.004	0.020	0.010	0.03	75
	9	23.2	7.2	134	65	38	22	9.7	146	0.67	0.009	1.200	0.055	1.26	30
	10	20.7	7.6	777	357	369	0	16.2	233	0.55	0.176	8.800	0.140	9.12	35
	11	15.9	6.2	88	36	20	24	7.1	96	0.30	0.010	1.250	0.065	1.33	75
	12	16.3	5.8	58	24	10	9	7.2	98	0.36	0.005	0.750	0.000	0.76	60
	13	18.5	5.9	77	34	9	37	7.0	99	0.84	0.015	0.050	0.060	0.12	70
	14	16.4	6.5	96	40	26	38	7.3	99	0.83	0.018	0.140	0.105	0.26	65
II Dry season	1	13.8	7.5	432	170	152	18	8.3	105	0.24	0.015	0.025	0.010	0.05	85
	2	17.4	7.5	878	375	168	24	8.5	115	0.30	0.015	0.030	0.000	0.04	75
	3	18.5	7.7	857	376	168	13	8.7	119	0.23	0.016	0.025	0.000	0.04	75
	4	18.5	7.2	61	27	14	25	8.3	114	0.25	0.015	0.020	0.000	0.04	65
	5	20.5	6.8	79	36	16	19	9.3	131	0.28	0.016	0.020	0.015	0.05	70
	6	17.4	5.8	42	18	7	16	7.9	106	0.29	0.014	0.020	0.010	0.04	65
	7	25.6	7.4	71	36	21	14	9.2	144	0.26	0.016	0.030	0.035	0.08	50
	8	22.7	5.5	53	25	14	17	8.3	123	0.28	0.017	0.030	0.000	0.05	65
	9	14.7	6.1	283	113	83	0	4.2	54	0.32	0.016	0.030	0.000	0.05	30
II Dry season	10	18.8	7.4	969	427	461	0	9.7	134	0.83	0.018	0.030	0.015	0.06	35
	11	9.9	6.4	279	99	91	0	6.1	71	0.28	0.015	0.025	0.000	0.04	75
	12	13.8	5.8	94	37	17	0	6.2	81	0.26	0.016	0.030	0.025	0.07	60
	13	12.0	6.3	129	48	22	0	6.4	80	0.26	0.017	0.030	0.015	0.06	60
	14	19.5	6.9	172	77	65	23	6.9	99	0.24	0.014	0.020	0.010	0.04	75
III Rainy season	1	16.5	7.7	125	53	41	32	5.3	71	0.38	0.007	0.015	0.040	0.06	75
	2	16.0	7.2	306	127	66	43	6.1	80	0.29	0.025	0.040	0.145	0.21	65
	3	18.0	7.7	313	136	72	68	6.1	82	0.29	0.017	0.025	0.115	0.16	75
	4	23.9	6.8	40	20	10	31	5.2	79	0.29	0.006	0.010	0.010	0.03	65
	5	26.1	7.9	65	33	19	27	5.5	86	0.31	0.005	0.010	0.005	0.02	70
	6	17.1	5.1	42	18	4	80	5.9	79	0.36	0.006	0.010	0.010	0.03	75
	7	19.9	5.3	55	25	16	62	5.2	73	0.27	0.011	0.020	0.055	0.09	50
	8	22.1	5.5	48	22	12	51	5.1	75	0.13	0.008	0.020	0.030	0.06	65
	9	24.2	6.8	138	68	56	36	5.1	77	0.50	0.005	0.010	0.015	0.03	30
	10	20.8	7.1	850	391	430	0	4.7	67	0.47	0.051	0.165	0.040	0.26	35
	11	15.2	6.8	91	37	34	18	5.0	66	0.71	0.010	0.030	0.140	0.18	65
	12	15.7	5.4	54	22	8	32	5.6	75	0.43	0.005	0.005	0.020	0.03	60
	13	15.8	5.9	78	32	15	45	5.9	80	0.55	0.010	0.030	0.015	0.05	70
	14	17.6	6.5	99	42	32	50	5.3	73	0.46	0.008	0.010	0.020	0.04	65

**Figure 145.**
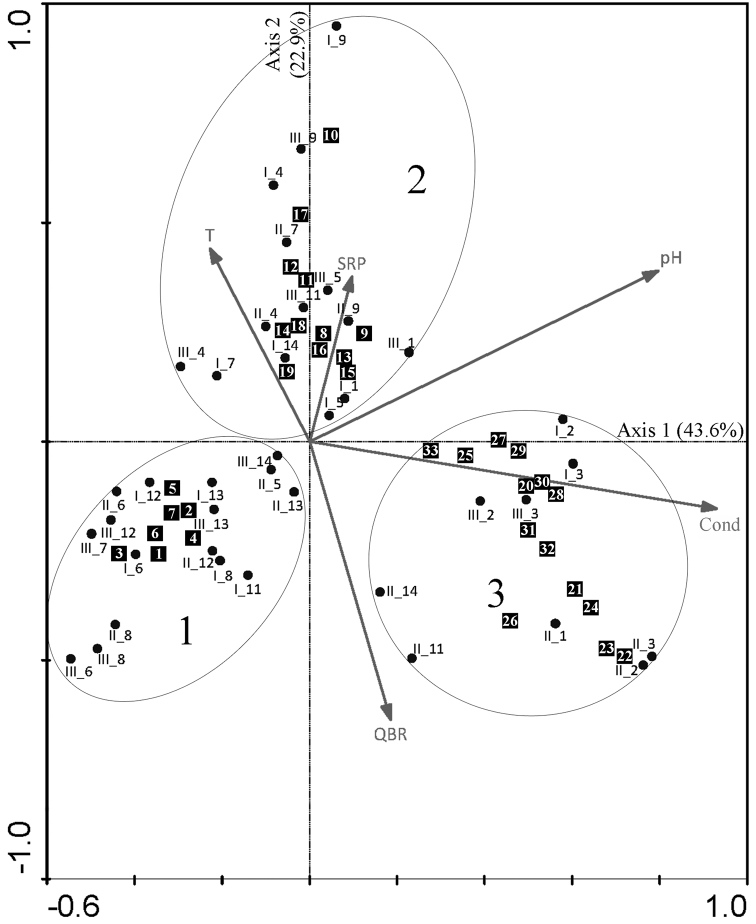
Canonical Correspondence Analysis (CCA) ordination plot. Distribution of sampling sites based on diatom abundance data in relation to statistically significant environmental variables. Three groups of samples are depicted within ovals. For visualization purposes, only species with significant IndVals (*p*< 0.05) are included in the plot. Black squares correspond to species; numbers within the black squares refer to taxa names in Table 4. Sampling sites are codified as follows: a Roman numeral indicating the sampling campaign (I, II and III), followed by an underscore symbol and an Arabic numeral indicating the sampling site (sites 1 to 14). For abbreviations and units of the physical and chemical parameters refer to Table 2.

On the CCA biplot three groups of samples were visualized (Fig. 145). The first group, situated at the bottom left part of the plot is composed of sites with the most acidic waters and lowest specific conductivity on average. The average number of species for this group was 16 (Table 3). This group was characterized by *Achnanthidium* sp. 1, the only taxon with a high indicator value (IndVal >50%). Other indicator taxa (IndVal 20–50%) for this group were Achnanthidium aff. catenatum (J.Bílý & Marvan) Lange-Bertalot, *Brachysira altepetlensis*, *Eunotia* sp. 3, *Fragilaria austriaca* (Grunow) Lange-Bertalot, *Frustulia crassinervia* (Brébisson) Lange-Bertalot & Krammer and *Gomphonema exilissimum* (Grunow) Lange-Bertalot & E. Reichardt (Table 4).

**Table 3.** Diversity indices and physical and chemical composition of the three groups visualized after the CCA. The mean value and standard deviation is provided for each variable. *S*= species richness; *H*’= Shannon-Wiener diversity index; *J*’ = Pielou evenness index. For abbreviations and units of the physical and chemical variables refer to Table 2.

	Group 1	Group 2	Group 3
*S*	16±5	21±6	17±4
*H*’	2.43±0.33	2.75±0.40	2.53±0.30
*J*’	0.61±0.12	0.63±0.16	0.56±0.17
T	18.2 ± 3.5	21 ± 4.8	16 ± 2.9
pH	5.9 ± 0.5	7 ± 0.5	7.3 ± 0.5
Cond	70 ± 24	104 ± 59	453 ± 249
TDS	30 ± 9	47 ± 23	191 ± 110
TA	14 ± 7	30 ± 20	107 ± 43
*v*	31 ± 23	24 ± 11	29 ± 20
DO	6.6 ± 1.2	6.6 ± 1.7	7.3 ± 1.1
DOS	91 ± 18	95 ± 27	97 ± 16
SRP	0.38 ± 0.17	0.5 ± 0.25	0.37 ± 0.23
NO_2_^‒^‒N	0.010 ± 0.005	0.008 ± 0.005	0.014 ± 0.006
NO_3_^‒^‒N	0.14 ± 0.35	0.11 ± 0.32	0.02 ± 0.01
NH_4_^+^‒N	0.022 ± 0.021	0.031 ± 0.043	0.039 ± 0.054
DIN	0.18 ± 0.35	0.15 ± 0.33	0.08 ± 0.06
QBR	66 ± 7	58 ± 18	75 ± 5

**Table 4.** Indicator taxa from the three groups visualized after the CCA. The indicator value of the taxa is accompanied by their relative abundance (RA) and relative frequency (RF) values. Significant IndVals (*p*< 0.05) are indicated in bold.

Taxa		Group 1			Group 2			Group 3		
		RA	RF	IndVal	RA	RF	IndVal	RA	RF	IndVal
1	*Achnanthidium* sp. 1	99	69	**68**	0	7	2	1	11	0
2	Achnanthidium aff. catenatum	77	63	**48**	22	29	6	1	11	0
3	*Brachysira altepetlensis*	96	44	**42**	4	36	1	0	0	0
4	*Eunotia* sp. 3	99	31	**31**	1	7	0	0	0	0
5	*Fragilaria austriaca*	71	56	**40**	29	7	2	0	0	0
6	*Frustulia crassinervia*	100	31	**31**	0	0	0	0	0	0
7	*Gomphonema exilissimum*	64	75	**47**	31	43	13	5	22	1
8	*Craticula molestiformis*	16	31	5	74	79	**58**	10	33	3
9	*Craticula subminuscula*	3	6	0	84	57	**48**	13	56	7
10	*Cyclotella meneghiniana*	0	0	0	100	21	**21**	0	0	0
11	*Encyonema minutum*	11	13	1	85	64	**54**	4	11	0
12	*Eolimna* sp. 1	12	13	2	88	36	**31**	0	0	0
13	*Fistulifera saprophila*	4	6	0	79	57	**45**	17	22	4
14	Gomphonema aff. sarcophagus	3	13	0	96	43	**41**	1	11	0
15	*Mayamaea permitis*	9	31	3	69	86	**59**	22	67	15
16	*Navicula rostellata*	2	6	0	87	57	**50**	11	11	1
17	*Nitzschia gracilis*	0	0	0	100	29	**29**	0	0	0
18	Nitzschia palea var. debilis	8	25	2	91	50	**45**	1	11	0
19	Nitzschia palea var. tenuirostris	9	38	4	91	64	**58**	0	0	0
20	*Amphora pediculus*	0	0	0	3	7	0	97	44	**43**
21	*Cocconeis* sp. 2	0	0	0	2	14	0	98	67	**66**
22	*Cocconeis pediculus*	0	0	0	0	0	0	100	22	**22**
23	*Epithemia adnata*	0	0	0	0	0	0	100	33	**33**
24	*Epithemia sorex*	0	0	0	4	7	0	96	44	**43**
25	*Gomphonema pumilum*	0	6	0	28	21	6	72	67	**48**
26	*Gomphonema minusculum*	0	0	0	0	0	0	100	33	**33**
27	*Halamphora montana*	0	0	0	16	14	2	84	56	**46**
28	*Navicula reichardtiana*	0	0	0	0	0	0	100	56	**56**
29	*Navicula gregaria*	0	0	0	13	14	2	87	56	**48**
30	*Nitzschia inconspicua*	1	13	0	0	0	0	99	56	**55**
31	*Planothidium victori*	0	6	0	12	29	3	88	78	**69**
32	*Reimeria sinuata*	0	0	0	0	0	0	100	67	**67**
33	*Sellaphora atomoides*	10	31	3	20	29	6	70	78	**54**

The second group, found on the upper middle side of the plot contains samples with circumneutral waters, low in specific conductivity and the highest mean temperature and soluble reactive phosphorous concentrations. The mean number of species was 17 (Table 3). These sites were characterized by *Craticula molestiformis* (Hustedt) Mayama, *Encyonema minutum* (Hilse) D.G. Mann, *Mayamaea permitis* (Hustedt) Bruder & Medlin and Nitzschia palea var. tenuirostris Grunow, all these taxa with high and significant IndVals (>50%) (Table 4).

Samples from the third group correspond to well mineralized waters with the highest pH values on average, and also the lowest nitrogen concentrations. The sites in this group scored the higher values for the QBR on average. The mean species richness was 17 (Table 3). This group was characterized by *Cocconeis* sp. 2, *Navicula reichardtiana* Lange-Bertalot, *Nitzschia inconspicua* Grunow, *Planothidium victori* Novis, Braidwood & Kilroy, *Reimeria sinuata* (W. Gregory) Kociolek & Stoermer and *Sellaphora atomoides* (Grunow) C.E. Wetzel & Van de Vijver.

The three sampling campaigns of eight sites are within the same groups of the CCA plot (Fig. 145), pointing out to stability of the diatom communities: samples from sites 6, 8, 12 and 13 are within group 1; sites 4 and 9 within group 2; and sites 2 and 3 in group 3.

In contrast, in 5 sites there were changes of the samples among the three groups. For site 7, one sample from the rainy season is together with the sample from the dry season in group 2, whereas the other rainy season sample is in group 1. The three samples of sites 11 and 14 are one in each of the three different groups observed in the CCA plot (Fig. 145). Only in sites 1 and 5, both rainy season samples are together within the same group, whereas the samples of the dry season are located in a different group.

## Discussion

*Species composition and taxonomy.* The species richness found, 274 taxa, was relatively high compared to previous studies on the basin: 209 taxa were found by Abarca-Mejía (2010) from 59 samples analyzed from three substrates; 178 taxa by Segura-García (2012) from 66 epilithon samples analyzed; 173 taxa by Mora et al. (2015) from 12 epilithon samples; and 70 taxa by Segura-García (2016) from 16 epilithon samples. This kind of comparison is difficult to make since it depends on the number of samples analyzed, the timing of the samplings, the physical and chemical composition of the waters, the number of substrates sampled and the taxonomic effort with which the diatom valves were analyzed (Morales et al. 2001, Veselá and Johansen 2009). Nevertheless, our results on taxa diversity are higher than the four previous studies conducted in the basin.

The resulting high diversity found in our study can be explained by the detail at which samples were analyzed under both LM and SEM, which resulted in the separation of several morphodemes instead of lumping them into species complexes. The fact that a third of the flora, 94 morphodemes, could not be assigned to described species is not surprising due to the nature of the samples, coming from within the tropics, for which no extensive identification floras have been produced yet, compared to northern temperate regions. Furthermore, it is encouraging to have such a big number of unidentified morphodemes, because they could be helpful in the quest of unravelling if the freshwater diatom floras of Mexico have certain biogeographical affinities, as it would be expected due to the fact that the country lies within the so called Mexican Transition Zone, a complex area in which Neotropical and Nearctic biotic elements converge (Huidobro et al. 2006). This task could be facilitated by coupling detailed morphological examinations with molecular tools (Trobajo et al. 2009, Abarca et al. 2014, Zimmermann et al. 2014).

In most of the freshwater diatom floras generated for Mexico, there seems to be a high intrinsic cosmopolitism, with a large proportion of taxa from north temperate waters. Nowadays it seems unlikely to find large amounts of shared species with north temperate regions due to mounting evidence that even microorganisms like diatoms have biogeography (Kociolek and Spaulding 2000, Vanormelingen et al. 2008, Abarca et al. 2014). This raises the question of identification literature and the detail with which samples are analyzed, such as force-fitting identifications to north temperate taxa and lumping into broad species complexes due to limited high resolution microscopy tools.

On the other hand, finding a large proportion of cosmopolitan taxa should not be that surprising since isolated areas such the Andes have shown to have as much as 42% cosmopolitan taxa, but also a considerable proportion of newly described taxa (9.5%) plus seemingly endemic regionals (Lange-Bertalot 2007). So far these 9.5% of newly described taxa have not been the case for the flora from the Lerma-Chapala Basin or even Central Mexico, for which no species from streams have been described as new in the last 25 years. Within the basin, the number of unidentified taxa, potentially containing undescribed species varies from 6% in Segura-García (2012), to 19% in Abarca-Mejía (2010) and 22% in Mora et al. (2015) but as those authors pointed out, further examinations on some of those taxa are needed to determine if they really should be described as new species.

Another hypothesis that could explain the high species richness found in our study is the heterogeneity of environmental conditions of the study areas: a) the sampling campaigns were done in both rainy and dry seasons; b) varied geomorphologies of the streams from headwaters to the midlands and also from the plains, resulting in different riparian communities, reflected in the QBR index values obtained; c) streams ranging from perennial to temporary; d) heterogeneity of physical and chemical composition of the water. Environmental heterogeneity of habitats has been proposed in other studies as a determinant of species richness and distribution (Petrov and Nevrova, 2014).

An additional indicator of the heterogeneity of the studied sites is the fact that no single taxon was found in all samples, which contrast with previous findings on the Lerma-Chapala Basin, where the following taxa were found in all sites and seasons *Craticula subminuscula*, *Gomphonema parvulum*, *Navicula veneta*, *Nitzschia amphibia*, *N. capitellata*, *N. palea* and *Sellaphora pupula* (Segura-García 2012, Mora et al. 2015).

When looking at the macroalgae of the studied streams, it is worth mentioning that sampling sites 11–14 host red algae like *Batrachospermum gelatinosum* (Linnaeus) De Candolle, *Paralemanea mexicana* (Kützing) Vis & Sheath and *Sirodotia suecica* Kylin, species typically found in headwater mountain streams of temperate regions (Bojorge-García et al. 2010). On the other hand, in sites 1–10 species rather associated to warmer waters were found, such as *Cladophora mexicana* P. Crouan & H. Crouan. This is another indicator of the heterogeneity of the sampling sites.

*Diatom communities.* The different diatom compositions found in the Lerma-Chapala Basin were mainly driven by specific conductivity and pH. Temperature, soluble reactive phosphorous and the Riparian Forest Quality Index were statistically significant but when analyzing the mean values and their standard deviations, the border between each group was not distinct.

For both specific conductivity and pH, the lowest values were recorded in the streams located in the headwaters, which is logical since water there has not gone deep into the geological matrix and therefore is not well mineralized. On the other hand, the higher values for both specific conductivity and pH were recorded on the midland and plains, where the streams received more contributions of well mineralized waters, for example from springs. There is no better example of this than what was recorded at sampling site 10, where pH values were high and specific conductivity values were the highest recorded for this study. This phenomenon is shown by Mahlknecht et al. (2004) in an aquifer recharge model for the same area where sampling sites 1–10 from our study are located. In the model, rain water normally has a pH of 5 but as water goes through the geological matrix it can reach pH values of up to 9 through several mineral dissolution processes and cation exchange, before it appears again at the surface i.e. springs.

No clear seasonal effect (rainy and dry seasons) was observed on the three groups of sampling sites observed after the CCA because in every group there are samples from both rainy seasons together with the dry season. Even though there were seasonal variations in physical and chemical factors such as specific conductivity, pH and water velocity, the community composition (species richness and abundance) apparently did not respond to those seasonal fluctuations (Rothfritz et al. 1997, Bojorge-García et al. 2014). This is well exemplified by the fact that the three samples of eight out of 13 sites included in the CCA remained within the same group during the three sampling periods, showing an overall stability of the diatom communities. This stability can be attributed to the fact that seasonal changes, e.g. in water velocity, discharge and chemical variables do not have long term effects so communities revert to their pre-disturbance state after the disappearance of the perturbation (Connell and Sousa 1983, Soininen and Eloranta 2004). On the other hand, perturbations such as mine tailings spills can have long lasting effects on diatom communities due to heavy metal pollution (Sabater 2000). The time it takes for communities to revert to a pre-disturbance state will largely depend on life span, reproduction and recolonization rates of the organisms as well as on the magnitude of the perturbation (Townsend et al. 1997, Soininen and Eloranta 2004). In order to relate seasonal changes in the community structure to fluctuations in environmental conditions, the timing and scale at which samplings should be made has to be proportional to the life span of the organism in question and cover a complete turnover of all individuals or longer (Soininen and Eloranta 2004). Since diatoms have short life cycles, high reproduction rates and recolonization rates that are within weeks (Round 1991, Licursi and Gómez 2009, Lowe 2011), it should be necessary to conduct intensive samplings to demonstrate dependency of changes in community structure due to fluctuations in environmental factors. This could be a reason why we observed an overall stability of the diatom communities. On the other hand, there were changes in the samples from 5 sampling sites, which can be attributed to the timing, since at the time of sampling the community composition was representing the changes due to seasonal fluctuations and not in an overall stable state after reverting from a perturbation (e.g. major flood, drought).

Regarding the characteristic species of the three groups visualized after the CCA, there are several similarities with previous reports on the ecological preferences of these taxa. Some species were found in all three groups but with varying relative abundances, so only those with the largest abundances were taken as the representative for a group.

For group 1, species from genera such as *Brachysira*, *Eunotia* and *Frustulia* are well regarded as characteristic from acidic and electrolyte poor waters (van Dam et al. 1994; Wolfe and Kling 2001; Hofmann et al. 2013; Vouilloud et al. 2014), which fits well to the chemical composition of the waters from the sites of this group. *Fragilaria austriaca*, *Frustulia crassinervia* and *Gomphonema exilissimum* are also regarded as indicators of low nutrients (van Dam et al. 1994). It is interesting to notice the presence of three taxa with uncertain identity, namely Achnanthidium aff. catenatum, *Achnanthidium* sp. 1 and *Eunotia* sp. 3, characteristic taxa of this group, which hints at the possibility to regard them as characteristic of acidic, and electrolyte and nutrient poor waters. But before their taxonomic position is confirmed, no comparisons about ecological preferences can be made.

The representative species from group 2 were taxa well regarded as indicators of circumneutral and eutrophic waters with varying degrees of perturbation such as *Craticula molestiformis*, *Mayamaea permitis* and N. palea var. tenuirostris (van Dam et al. 1994; Besse-Lototskaya et al. 2011; Hofmann et al. 2013). Other representatives of the beforehand conditions include *Craticula subminuscula* (Manguin) C.E. Wetzel & Ector, *Cyclotella meneghiniana* Kützing, *Fistulifera saprophila* (Lange-Bertalot & Bonik) Lange-Bertalot and *Navicula rostellata* Kützing (van Dam et al. 1994; Besse-Lototskaya et al. 2011; Hofmann et al. 2013). The exception for group 2 is *Encyonema minutum*, normally reported from oligo-mesotrophic waters, but the precise ecological preference of this taxon is difficult to tell since it has been long confounded with *Encyonema silesiacum* (Bleisch) D.G. Mann (Hofmann et al. 2013). In the sampling sites belonging to this group, the highest average phosphorous concentrations were recorded. Regarding the degree of perturbation, the QBR values for these sites scored the lowest values on average, which were related to human perturbation on the riparian forest. Some of these sites are in fact close to diffuse pollution sources such as cattle grazing and agriculture.

Regarding group 3, its characteristic species also confirm the meso-eutrophic, mineralized and alkaliphilous nature of its waters, with taxa such a *Cocconeis* sp. 2 (*C. placentula* Ehrenberg *sensu lato* based only on LM observations), *Navicula reichardtiana*, *Nitzschia inconspicua*, *Planothidium victori* (formerly within *Planothidium frequentissimum* (Lange-Bertalot) Lange-Bertalot *sensu lato*), *Reimeria sinuata* and *Sellaphora atomoides* (former *Eolimna tantula* (Hustedt) Lange-Bertalot) (van Dam et al. 1994; Lange-Bertalot 2001). Other taxa characteristic of this conditions include *Amphora pediculus* (Kützing) Grunow, *Epithemia adnata* (Kützing) Brébisson, *Epithemia sorex* Kützing, *Gomphonema pumilum* (Grunow) E. Reichardt & Lange-Bertalot, *Halamphora montana* (Krasske) Levkov and *Navicula gregaria* Donkin (van Dam et al. 1994; Lange-Bertalot 2001). When looking at the average dissolved inorganic nitrogen from the group, the lowest of all three groups, it is hard to explain it based on the seasonal inputs from the surrounding environment. But when looking at the algae present on the water, it is worth mentioning that on all of the sites from this group *Nostoc* spp. was found, in some cases blooming. The presence of these nitrogen-fixing cyanobacteria is regarded as an indicator of poor nitrogen concentrations since these algae can thrive under this condition by actively fixating atmospheric nitrogen (Grimm and Petrone 1997).

## Conclusion

This work contributed to increase the knowledge of the diatom flora from the Lerma-Chapala Basin, Central Mexico, providing a diversity baseline and evidence of its distinctiveness from the floras of other areas in Mexico, with a large proportion of unidentified taxa to be described as new. The studied diatom communities are subjected to moderate environmental disturbance, representing a transition between warm and cold waters, with ionic composition, temperature and the quality of the riparian forest being the main factors defining the community composition observed. The next approach to investigate the diatom diversity of the region would be by means of environmental DNA metabarcoding in combination with the development of a taxonomic reference database, in order to highlight the complementary aspect of classical taxonomy and eDNA metabarcoding, i.e. the importance of the reciprocal illumination (Visco et al. 2015; Zimmermann et al. 2015).

## Supplementary Material

XML Treatment for Brachysira altepetlensis

XML Treatment for Sellaphora queretana

XML Treatment for Sellaphora rhombica

## Supplementary materials

Supplementary material 1 Diatom taxa list from the Lerma-Chapala Basin, Central Mexico and identification references Data type: species data Explanation note: Diatom taxa list from the Lerma‒Chapala Basin, Central Mexico This dataset is made available under the Open Database License (http://opendatacommons.org/licenses/odbl/1.0/). The Open Database License (ODbL) is a license agreement intended to allow users to freely share, modify, and use this Dataset while maintaining this same freedom for others, provided that the original source and author(s) are credited.Demetrio Mora, Javier Carmona, Regine Jahn, Jonas Zimmermann, Nélida Abarca
