# Supplementary material for: Epilithic diatom communities of selected streams from the Lerma-Chapala Basin, Central Mexico, with the description of two new species
Source: PhytoKeys. 2017 Oct 11;(88):39–69. doi: 10.3897/phytokeys.88.14612 (PMC5672134; doi:10.3897/phytokeys.88.14612)
Supplement: Supplementary material 1 — Diatom taxa list from the Lerma-Chapala Basin, Central Mexico and identification references [file phytokeys-88-039-s001.docx]

Supplementary file 1. Diatom taxa list from the Lerma‒Chapala Basin, Central Mexico. Taxa names are accompanied by the identification reference as well as the plate and figure(s) matching our observations in the references. Online identification references from the Diatoms of the United States website do not include plate or figure number. The complete citation of the identification reference is given at the end of the taxa list.

* Indicates new record for the Lerma‒Chapala Basin.

| **Taxa** | **Identification reference** | |
| --- | --- | --- |
| *Achnanthes inflata* var. *inflata* (Kützing) Grunow | Metzeltin et al. 2005 | Pl. 28: 2 |
| *Achnanthidium* aff. *catenatum* (J.Bílý et Marvan) Lange-Bertalot | Hofmann et al. 2013 | Pl. 23: 69‒72 |
| *Achnanthidium exiguum* (Grunow) Czarnecki | Rumrich et al. 2000 | Pl. 27: 14‒15 |
| ** Achnanthidium exile* (Kützing) Round et Bukhtiyarova | Hofmann et al. 2013 | Pl. 23: 14 |
| *Achnanthidium minutissimum* (Kützing) Czarnecki | Hofmann et al. 2013 | Pl. 23: 18‒19 |
| *Achnanthidium* sp. 1 |  |  |
| *Achnanthidium* sp. 2 |  |  |
| *Achnanthidium* sp. 3 |  |  |
| *Achnanthidium* sp. 4 |  |  |
| *Achnanthidium* sp. 5 |  |  |
| *Achnanthidium* sp. 6 |  |  |
| *Amphora pediculus* (Kützing) Grunow | Levkov 2009 | Pl. 78: 40‒47 |
| ** Brachysira altepetlensis* D. Mora, R. Jahn et N. Abarca | This study |  |
| ** Brachysira brebissonii* Ross | Lange-Bertalot and Moser 1994 | Pl. 41: 17 |
| ** Brachysira microcephala* (Grunow) Compère | Hamilton 2010 |  |
| *Brachysira* sp. 1 |  |  |
| *Brachysira* sp. 2 |  |  |
| *Caloneis bacillum* (Grunow) Cleve | Hofmann et al. 2013 | Pl. 67: 28 |
| ** Caloneis clevei* var. *uruguayensis* Frenguelli | Metzeltin et al. 2005 | Pl. 154: 7‒8 |
| *Caloneis schumanniana* (Grunow) Cleve | Stancheva et al. 2009 | Fig. 24 |
| ** Caloneis silicula* (Ehrenberg) Cleve | Metzeltin et al. 2005 | Pl. 154: 23 |
| *Caloneis* cf. *silicula var. elliptica* Frenguelli | Metzeltin et al. 2005 | Pl. 155: 21 |
| *Caloneis* sp. 1 |  |  |
| *Caloneis* sp. 2 |  |  |
| *Caloneis* sp. 3 |  |  |
| *Caloneis* sp. 4 |  |  |
| * *Chamaepinnularia submuscicola* (Krasske) Lange-Bertalot | Hofmann et al. 2013 | Pl. 50: 35‒36 |
| cf. *Chamaepinnularia* sp. |  |  |
| *Cocconeis pediculus* Ehrenberg | Jahn et al. 2009 | P. 281: 10 and 15 |
| *Cocconeis* sp. 1 |  |  |
| *Cocconeis* sp. 2 |  |  |
| *Craticula accomoda* (Hustedt) D.G. Mann | Lange-Bertalot 2001 | Pl. 93: 3 |
| * *Craticula acidoclinata* Lange-Bertalot et Metzeltin | Lange-Bertalot 2001 | Pl. 87: 1 |
| *Craticula ambigua* (Ehrenberg) D.G. Mann | Lange-Bertalot 2001 | Pl. 82: 4 |
| * *Craticula buderi* (Hustedt) Lange-Bertalot | Lange-Bertalot 2001 | Pl. 90: 22 |
| *Craticula molestiformis* (Hustedt) Mayama | Krammer and Lange-Bertalot 1997a | Pl. 45: 7‒9 |
| *Craticula* cf. *pumilio* Lange-Bertalot et U.Rumrich | Metzeltin et al. 2005 | Pl. 96: 7 |
| *Craticula subminuscula* (Manguin) C.E. Wetzel et Ector | Rumrich et al. 2000 | Pl. 72: 8‒9 |
| * *Craticula submolesta* (Hustedt) Lange-Bertalot | Lange-Bertalot 2001 | Pl. 93: 31‒34 |
| *Craticula* sp. 1 |  |  |
| *Craticula* sp. 2 |  |  |
| *Cyclostephanos invisitatus* (Hohn et Hellermann) Theriot, Stoermer et Håkasson | Håkansson 2002 | Fig. 221 |
| *Cyclotella atomus* Hustedt | Krammer and Lange-Bertalot 1991a | Pl. 51: Fig. 19 |
| *Cyclotella meneghiniana* Kützing | Krammer and Lange-Bertalot 1991a | Pl. 44: 1‒2, 4 and 9 |
| *Cymbella kolbei* Hustedt | Krammer 2002 | Pl. 14: 19‒21 |
| ** Cymbella tropica* Krammer et Metzeltin | Krammer 2002 | Pl. 44: 2 |
| ** Cymbopleura naviculiformis* (Auerswald) Krammer | Hofmann et al. 2013 | Pl. 83: 20‒23 |
| *Diadesmis confervacea* Kützing | Metzeltin et al. 2005 | Pl. 68: 12‒16 |
| ** Discostella stelligera* (Cleve et Grunow) Houk et Klee | Krammer and Lange-Bertalot 1991a | Pl. 49: 3 |
| ** Encyonema* *brevicapitatum* Krammer | Krammer 1997a | Pl. 34: 1‒7 |
| *Encyonema* cf. *hebridiforme* Krammer | Krammer 1997a | Pl. 11: 16 |
| ** Encyonema jemtlandicum* Krammer | Krammer 1997a | Pl. 35: 1‒5 |
| ** Encyonema jemtlandicum* var. *venezolanum* Krammer | Krammer 1997a | Pl. 14: 4 |
| ** Encyonema* *minutiforme* Krammer | Krammer 1997a | Pl. 18: 12 and 14 |
| *Encyonema* cf. *minutiforme* Krammer | Krammer 1997a | Pl. 18: 12 and 14 |
| ** Encyonema minutum* (Hilse) D.G. Mann | Krammer 1997a | Pl. 25: 5‒10, 16‒19 |
| ** Encyonema pergracile* Krammer | Krammer 1997a | Pl. 88: 6 |
| *Encyonema silesiacum* (Bleisch) D.G. Mann | Krammer 1997a | Pl. 4: |
| *Encyonema triangulum* (Ehrenberg) Kützing | Krammer 1997a | Pl. 78: 1 and 5 |
| *Encyonema* sp. 1 |  |  |
| ** Encyonopsis subminuta* Krammer et E.Reichardt | Krammer 1997b | Pl. 144: 6‒11 |
| *Encyonopsis* cf. *thienemannii* (Hustedt) Krammer | Krammer 1997b | Pl. 149: 28‒29 |
| *Encyonopsis* sp. 1 |  |  |
| *Eolimna* sp. 1 |  |  |
| *Eolimna* sp. 2 |  |  |
| *Eolimna* sp. 3 |  |  |
| *Eolimna* sp. 4 |  |  |
| *Epithemia adnata* (Kützing) Brébisson | Metzeltin et al. 2005 | Pl. 190: 8‒11 |
| *Epithemia sorex* Kützing | Levkov et al. 2007 | Pl. 198: 13‒15 |
| *Epithemia turgida* (Ehrenberg) Kützing | Hofmann et al. 2013 | Pl. 120: 1 |
| ** Eunotia* *bidens* Ehrenberg | Lange-Bertalot et al. 2011 | Pl. 80: 2 |
| *Eunotia* cf. *bigibba* var. *pumila* Grunow | Metzeltin and Lange-Bertalot 2007 | Pl. 90: 12 |
| * *Eunotia kruegeri* Lange-Bertalot | Werum and Lange-Bertalot 2004 | Pl. 5: 8 |
| *Eunotia* *major* var. *major* (W. Smith) Rabenhorst | Metzeltin et al. 2005 | Pl. 17: 4‒5 |
| *Eunotia* cf. *meridiana* Metzeltin et Lange-Bertalot | Metzeltin et al. 2005 | Pl. 20: 22‒28 |
| * *Eunotia metamonodon* Lange-Bertalot | Lange-Bertalot et al. 2011 | Pl. 217: 4‒5 |
| ** Eunotia* *minor* (Kützing) Ehrenberg | Hofmann et al. 2013 | Pl. 15: 5‒6 |
| *Eunotia* cf. *monodon* Ehrenberg | Metzeltin et al. 2005 | Pl. 19: 18 |
| * *Eunotia mucophila* (Lange-Bertalot, Nörpel-Schempp et Alles) Lange-Bertalot | Lange-Bertalot et al. 2011 | Pl. 23: 21‒23 |
| ** Eunotia* *tridentula* Ehrenberg | Metzeltin et al. 2005 | Pl. 21: 2‒6 |
| *Eunotia* sp. 1 |  |  |
| *Eunotia* sp. 2 |  |  |
| *Eunotia* sp. 3 |  |  |
| *Eunotia* sp. 4 |  |  |
| *Eunotia* sp. 5 |  |  |
| *Eunotia* sp. 6 |  |  |
| *Fallacia pygmaea* (Kützing) D.G. Mann | Metzeltin et al. 2005 | Pl. 51: 15‒18 |
| *Fistulifera saprophila* (Lange-Bertalot et Bonik) Lange-Bertalot | Lange-Bertalot 2001 | Pl. 111: 1 |
| * *Fragilaria austriaca* (Grunow) Lange-Bertalot | Hofmann et al. 2013 | Pl. 7: 33‒34 |
| *Fragilaria* *bidens* Heiberg | Krammer and Lange-Bertalot 1991a | Pl. 111: 21 |
| ** Fragilaria pectinalis* (O.F. Müller) Lyngbye | Wetzel and Ector 2015 | P. 280: 78‒80 |
| ** Fragilaria* *rumpens* (Kützing) Carlson | Hofmann et al. 2013 | Pl. 16‒17 |
| * *Fragilaria tenera* (W. Smith) Lange-Bertalot | Hofmann et al. 2013 | Pl. 7: 18‒19 |
| * *Frustulia crassinervia* (Brébisson) Lange-Bertalot et Krammer | Metzeltin and Lange-Bertalot 1998 | Pl. 117: 10 and 13 |
| *Frustulia neomundana* Lange-Bertalot et Rumrich | Rumrich et al. 2000 | Pl. 97: 5‒6 |
| *Frustulia* cf. *spicula* ssp. *spicula* Amossé | Lange-Bertalot 2001 | Pl. 138: 3‒4 |
| *Frustulia* cf. *undosa* Melzeltin et Lange-Bertalot | Metzeltin and Lange-Bertalot 1998 | Pl. 117: 6‒7 |
| *Frustulia vulgaris* (Thwaites) De Toni | Rumrich et al. 2000 | Pl. 96: 7 |
| *Geissleria decussis* (Østrup) Lange-Bertalot et Metzeltin | Metzeltin et al. 2005 | Pl. 91: 24‒25 |
| *Gomphonema acuminatum* Ehrenberg | Levkov et al. 2016 | Pl. 3: 1 |
| *Gomphonema affine* var. *affine* Kützing | Reichardt 1999 | Pl. 7: 2‒3 |
| *Gomphonema exilissimum* (Grunow) Lange-Bertalot et E.Reichardt | Levkov et al. 2016 | Pl. 127: 1‒28 |
| ** Gomphonema graciledictum* E. Reichardt | Levkov et al. 2016 | Pl. 44: 20 and 24 |
| *Gomphonema* aff. *graciledictum* E. Reichardt | Levkov et al. 2016 | Pl. 44: 20 and 24 |
| *Gomphonema innocens* E. Reichardt | Levkov et al. 2016 | Pl. 123: 11‒32 |
| *Gomphonema lagenula* Kützing | Levkov et al. 2016 | Pl. 102: 39‒47 |
| *Gomphonema* cf. *lagenula* Kützing | Levkov et al. 2016 |  |
| Gomphonema cf. lippertii E. Reichardt et Lange-Bertalot | Reichardt 1999 | Pl. 21: 6‒7 |
| *Gomphonema* aff. *mariovense* Levkov et Tofilovska | Levkov et al. 2016 | Pl. 110: 10 |
| *Gomphonema* *minusculum* Krasske | Levkov et al. 2016 | Pl. 161: 1‒25 |
| ** Gomphonema naviculoides* W. Smith | Reichardt 2015 | Fig. 26‒27 |
| *Gomphonema* cf. *naviculoides* W. Smith | Reichardt 2015 | Fig. 26‒27 |
| *Gomphonema parvulum* (Kützing) Kützing | Levkov et al. 2016 | Pl. 102: 1‒19 |
| * *Gomphonema parvuliforme* Levkov, Mitic-Kopanja et E.Reichardt | Levkov et al. 2016 | Pl. 105: 1‒34 |
| *Gomphonema* cf. *parvuliforme* Levkov, Mitic-Kopanja et E. Reichardt | Levkov et al. 2016 | Pl. 105: 1‒4 |
| *Gomphonema* aff. *parvulius* (Lange-Bertalot et E. Reichardt) Lange-Bertalot et E. Reichardt | Levkov et al. 2016 | Pl. 124: 42‒44 |
| *Gomphonema pumilum* (Grunow) E. Reichardt et Lange-Bertalot | Levkov et al. 2016 | Pl. 151: 36‒39 |
| *Gomphonema salae* Lange-Bertalot et E. Reichardt | Metzeltin and Lange-Bertalot 1998 | Pl. 157: 4 |
| *Gomphonema* aff. *sarcophagus* W. Gregory | Levkov et al. 2016 | Pl. 94: 13 |
| *Gomphonema stonei* E. Reichardt | Reichardt 1999 | Pl. 13: 12 |
| *Gomphonema subclavatum* (Grunow) Grunow | Hofmann et al. 2013 | Pl. 95: 23‒24 |
| *Gomphonema* sp. 1 |  |  |
| *Gomphonema* sp. 2 |  |  |
| *Gomphonema* sp. 3 |  |  |
| *Gomphonema* sp. 4 |  |  |
| *Halamphora montana* (Krasske) Levkov | Levkov 2009 | Pl. 93: 10‒12 |
| *Halamphora* cf. *pseudomontana* (Cholnoky) Levkov | Levkov 2009 | Pl. 104: 20 |
| *Halamphora veneta* (Kützing) Levkov | Levkov 2009 | Pl. 94: 17‒19 |
| *Hantzschia abundans* Lange-Bertalot | Metzeltin et al. 2005 | Pl. 212: 5‒6 |
| *Hantzschia amphioxys* (Ehrenberg) Grunow | Metzeltin et al. 2005 | Pl. 212: 10‒12 |
| *Hantzschia* sp. 1 |  |  |
| * *Humidophila contenta* (Grunow) Lowe, Kociolek, Johansen, Van de Vijver, Lange-Bertalot et Kopalová | Metzeltin et al. 2005 | Pl. 57: 13‒14 |
| *Humidophila* sp. |  |  |
| *Luticola* *goeppertiana* (Bleisch) D.G. Mann | Hofmann et al. 2013 | Pl. 45: 22 |
| *Luticola kotschyi* (Grunow) D.G. Mann | Rumrich et al. 2000 | Pl. 60: 14 |
| *Luticola* *mutica* (Kützing) D.G. Mann | Hofmann et al. 2013 | Pl. 45: 36‒37 and 39 |
| *Luticola* cf. *tomesii* Gerd Moser, Lange-Bertalot et Metzeltin | Levkov et al. 2013 | Pl. 25: 5‒7 |
| ** Luticola* *tropica* Levkov, Metzeltin et A. Pavlov | Levkov et al. 2013 | Pl. 196: 12 |
| ** Luticola undulata* (Hilse) D.G. Mann | Levkov et al. 2013 | Pl. 181: 43‒46 |
| *Luticola ventricosa* (Kützing) D.G. Mann | Hofmann et al. 2013 | Pl. 45: 41‒42 |
| *Luticola* sp. 1 |  |  |
| *Luticola* sp. 2 |  |  |
| *Mayamaea* cf. *crassistriata* Lange-Bertalot, Cavacini, Tagliaventi et Alfinito | Lange-Bertalot et al. 2003 | Pl. 17: 4‒5 |
| *Mayamaea permitis* (Hustedt) Bruder et Medlin | Lange-Bertalot 2001 | Pl. 104: 7‒13 |
| *Mayamaea* sp. 1 |  |  |
| *Navicula angusta* Grunow | Lange-Bertalot 2001 | Pl. 2: 5 |
| *Navicula antonii* Lange-Bertalot | Lange-Bertalot 2001 | Pl. 13: 1 and 8 |
| *Navicula capitatoradiata* H. Germain | Lange-Bertalot 2001 | Pl. 29: 17 |
| *Navicula cryptocephala* Kützing | Lange-Bertalot 2001 | Pl. 17: 4‒5 |
| *Navicula* cf. *cryptocephala* Kützing | Hofmann et al. 2013 | Pl. 31: 8 |
| *Navicula cryptotenella* Lange-Bertalot | Lange-Bertalot 2001 | Pl. 26: 25 |
| *Navicula erifuga* Lange-Bertalot | Lange-Bertalot 2001 | Pl. 35: 14‒16 |
| *Navicula germainii* Wallace | Lange-Bertalot 2001 | Pl. 35: 7‒9 |
| *Navicula* *gregaria* Donkin | Metzeltin et al. 2005 | Pl. 45: 16‒17 |
| *Navicula* cf. *hoffmanniae* Lange-Bertalot | Lange-Bertalot 2001 | Pl. 20: 21 |
| *Navicula libonensis* Schoeman | Lange-Bertalot 2001 | Pl. 43: 7‒10 |
| ** Navicula notha* Wallace | Lange-Bertalot 2001 | Pl. 40: 22‒28 |
| * *Navicula reichardtiana* Lange-Bertalot | Lange-Bertalot 2001 | Pl. 13: 25‒35 |
| *Navicula riediana* Lange-Bertalot | Lange-Bertalot 2001 | Pl. 34: 1 |
| *Navicula rostellata* Kützing | Lange-Bertalot 2001 | Pl. 35: 1‒6 |
| *Navicula symmetrica* R.M. Patrick | Lange-Bertalot 2001 | Pl. 39: 9‒12 |
| *Navicula* cf. *tenelloides* Hustedt | Lange-Bertalot 2001 | Pl. 32: 1 |
| *Navicula trivialis* Lange-Bertalot | Lange-Bertalot 2001 | Pl. 29: 1‒7 |
| *Navicula veneta* Kützing | Lange-Bertalot 2001 | Pl. 14: 23‒30 |
| *Navigiolum uruguayense* Metzeltin, Lange-Bertalot et García-Rodríguez | Metzeltin et al. 2005 | Pl. 44: 9‒10 |
| *Neidium* cf. *affine* (Ehrenberg) Pfitzer | Metzeltin et al. 2009 | Pl. 97: 12 |
| *Neidium ampliatum* (Ehrenberg) Krammer | Metzeltin and Lange-Bertalot 2007 | Pl. 189: 3 |
| ** Neidium amphigomphus* (Ehrenberg) Pfitzer | Metzeltin and Lange-Bertalot 2007 | Pl. 189: 2 |
| ** Neidium longiceps* (W. Gregory) R. Ross | Hofmann et al. 2013 | Pl. 53: 7 |
| *Neidium* sp. 1 |  |  |
| *Neidium* sp. 2 |  |  |
| *Nitzschia acicularis* (Kützing) W. Smith | Metzeltin et al. 2005 | Pl. 204: 12‒13 |
| *Nitzschia amphibia* Grunow | Metzeltin et al. 2005 | Pl. 207: 20‒33 |
| *Nitzschia* cf. *bacillum* Hustedt | Krammer and Lange-Bertalot 1997b | Pl. 78: 12 |
| *Nitzschia balcanica* Hustedt | Kociolek 2011a |  |
| *Nitzchia clausii* Hantzch | Krammer and Lange-Bertalot 1997b | Pl. 19: 3and 5 |
| *Nitzschia communis* Rabenhorst | Krammer and Lange-Bertalot 1997b | Pl. 79: 3‒4 |
| *Nitzschia desertorum* Hustedt | Krammer and Lange-Bertalot 1997b | Pl. 70: 10‒11 |
| *Nitzschia dissipata* var. *dissipata* (Kützing) Grunow | Krammer and Lange-Bertalot 1997b | Pl. 11: 2 |
| *Nitzschia fonticola* Grunow | Krammer and Lange-Bertalot 1997b | Pl. 75: 9, 13‒15 |
| *Nitzschia frustulum* (Kützing) Grunow | Krammer and Lange-Bertalot 1997b | Pl. 68: 1‒4 |
| *Nitzschia gracilis* Hantzsch | Krammer and Lange-Bertalot 1997b | Pl. 66: 7 |
| *Nitzschia* cf. *hantzschiana* Rabenhorst | Krammer and Lange-Bertalot 1997b | Pl. 73: 16‒17 |
| *Nitzschia inconspicua* Grunow | Krammer and Lange-Bertalot 1997b | Pl. 69: 4 |
| *Nitzschia intermedia* Hantzsch | Krammer and Lange-Bertalot 1997b | Pl. 61: 6‒8 |
| *Nitzschia lanceolata* W. Smith | Krammer and Lange-Bertalot 1997b | Pl. 16: 1 |
| *Nitzschia linearis* (Agardh) W. Smith | Metzeltin et al. 2005 | Pl. 206: 3‒4 |
| *Nitzschia palea* (Kützing) W. Smith | Hofmann et al. 2013 | Pl. 111: 1‒9 |
| *Nitzschia palea* var. *debilis* (Kützing) Grunow | Kociolek 2011b |  |
| *Nitzschia palea* var. *tenuirostris* Grunow | Kociolek 2011c |  |
| *Nitzschia paleacea* Grunow | Krammer and Lange-Bertalot 1997b | Pl. 81: 2‒5 |
| ** Nitzschia perminuta* (Grunow) Peragallo | Krammer and Lange-Bertalot 1997b | Pl. 72: 3‒4 |
| *Nitzschia recta* Hantzsch | Krammer and Lange-Bertalot 1997b | Pl. 12: 9 |
| *Nitzschia semirobusta* Lange-Bertalot | Lange-Bertalot 1993 | Pl. 120: 4‒8 |
| *Nitzschia sinuata* var. *delognei* (Grunow) Lange-Bertalot | Krammer and Lange-Bertalot 1997b | Pl. 40: 8 |
| *Nitzschia solita* Hustedt | Krammer and Lange-Bertalot 1997b | Pl. 71: 3 |
| ** Nitzschia sublinearis* Hustedt | Krammer and Lange-Bertalot 1997b | Pl. 58: 13 |
| *Nitzschia supralitorea* Lange-Bertalot | Krammer and Lange-Bertalot 1997b | Pl. 70: 17‒19 |
| ** Nitzschia tubicola* Grunow | Krammer and Lange-Bertalot 1997b | Pl. 63: 10 |
| *Nitzschia umbonata* (Ehrenberg) Lange-Bertalot | Krammer and Lange-Bertalot 1997b | Pl. 51: 1‒2 |
| *Nitzschia* sp. 1 |  |  |
| *Nitzschia* sp. 2 |  |  |
| *Nitzschia* sp. 3 |  |  |
| *Nitzschia* sp. 4 |  |  |
| *Nitzschia* sp. 5 |  |  |
| *Nitzschia* sp. 6 |  |  |
| ** Nupela praecipua* (E. Reichardt) E. Reichardt | Rumrich et al. 2000 | Pl. 33: 12 |
| * *Nupela wellneri* (Lange-Bertalot) Lange-Bertalot | Rumrich et al. 2000 | Pl. 35: 1‒3 |
| ** Pinnularia acrosphaeria* var. *parva* Krammer | Metzeltin and Lange-Bertalot 2007 | Pl. 276: 12 |
| ** Pinnularia anglica* morphodeme 1 Krammer | Krammer 2000 | Pl. 80: 7 and 11 |
| *Pinnularia anglica* morphodeme 2 Krammer | Krammer 2000 | Pl. 87: 3‒5 |
| *Pinnularia borealis* var. *borealis* Ehrenberg | Krammer 2000 | Pl. 7: 8 and 13 |
| ** Pinnularia borealis* var. *scalaris* (Ehrenberg) Rabenhorst | Krammer 2001 | Pl. 8: 11 |
| *Pinnularia* cf. *brebissonii* var. *acuta* Cleve-Euler | Krammer 2000 | Pl. 47: 5 |
| *Pinnularia divergens* W. Smith | Krammer 2000 | Pl. 29: 3‒4 |
| ** Pinnularia* *divergentissima* var. *divergentissima* Grunow | Krammer 2000 | Pl. 11: 7 |
| *Pinnularia gibba* Ehrenberg | Rumrich et al. 2000 | Pl. 140: 11 |
| ** Pinnularia* *mayeri* Krammer | Krammer 1992 | Pl. 42: 2 |
| *Pinnularia* cf. *meridiana* var. *parallela* Metzeltin et Krammer | Metzeltin and Lange-Bertalot 1998 | Pl. 181: 3 |
| ** Pinnularia parvulissima* Krammer | Krammer 2000 | Pl. 69: 10 |
| * *Pinnularia saprophila* Lange-Bertalot, H. Kobayasi et Krammer | Krammer 2000 | Pl. 85: 14‒18 |
| ** Pinnularia subbrevistriata* Krammer | Krammer 2000 | Pl. 70: 7‒8 |
| *Pinnularia* cf. *subcapitata* var. *elongata* Krammer | Krammer 1992 | Pl. 39: 2‒3 |
| *Pinnularia viridiformis* Krammer | Krammer 2000 | Pl. 161: 1 |
| *Pinnularia* sp. 1 |  |  |
| *Pinnularia* sp. 2 |  |  |
| *Pinnularia* sp. 3 |  |  |
| *Pinnularia* sp. 4 |  |  |
| *Pinnularia* sp. 5 |  |  |
| *Placoneis* cf. *constans* (Hustedt) E.J. Cox | Hofmann et al. 2013 | Pl. 48: 2 |
| *Placoneis undulata* (Østrup) Lange-Bertalot | Hofmann et al. 2013 | Pl. 47: 26 |
| *Planothidium incuriatum* C.E. Wetzel, Van de Vijver et Ector | Wetzel et al. 2013 | P. 49: 62‒67, 71‒73 |
| *Planothidium cryptolanceolatum* R. Jahn et N. Abarca | Jahn et al. 2017 | Figs 122‒146 |
| *Planothidium rostratum* (Østrup) Lange-Bertalot | Krammer and Lange-Bertalot 1991b | Pl. 43: 9‒11 |
| *Planothidium victori* Novis, Braidwood et Kilroy | Jahn et al. 2017 | Figs 272‒277 |
| *Pseudofallacia monoculata* (Hustedt) Liu, Kociolek et Wang | Metzeltin et al. 2005 | Pl. 61: 12 and 14 |
| *Reimeria sinuata* (W. Gregory) Kociolek et Stoermer | Rumrich et al. 2000 | Pl. 117: 14 |
| *Rhopalodia gibba* (Ehrenberg) O. Müller | Metzeltin et al. 2005 | 192: 3‒4 |
| *Rhopalodia operculata* (C. Agardh) Håkansson | Krammer and Lange-Bertalot 1997b | Pl. 115: 10 |
| *Sellaphora atomoides* (Grunow) C.E. Wetzel et Van de Vijver | Wetzel et al. 2015 | P. 220: 205‒235 |
| *Sellaphora bacilloides* Hustedt | Metzeltin et al. 2005 | Pl. 66: 19‒21 |
| * *Sellaphora blackfordensis* D.G. Mann et S. Droop | Hofmann et al. 2013 | Pl. 41: 11 |
| *Sellaphora cosmopolitana* (Lange-Bertalot) C.E. Wetzel et Ector | Rumrich et al. 2000 | Pl. 77: 36‒38 |
| *Sellaphora* cf. *elorantana* (Lange-Bertalot) C.E. Wetzel | Lange-Bertalot and Metzeltin 1996 | Pl. 28: 15‒17 |
| ** Sellaphora indistincta* Kociolek | Kociolek et al. 2014 | Pl. 54: 17 |
| *Sellaphora laevissima* (Kützing) D.G. Mann | Hofmann et al. 2013 | Pl. 41: 24 |
| ** Sellaphora madida* (Kociolek) C.E. Wetzel | Kociolek et al. 2014 | Pl. 28: 31‒34 |
| *Sellaphora nigri* (De Notaris) C.E. Wetzel et Ector | Wetzel et al. 2015 | P. 220: 319‒393 |
| *Sellaphora pupula* (Kützing) Mereschkowsky | Hofmann et al. 2013 | Pl. 41: 6‒10 |
| ** Sellaphora queretana* D. Mora, N. Abarca et J. Carmona | This study |  |
| *Sellaphora* cf. *rectangularis* (W. Gregory) Lange-Bertalot et Metzeltin | Lange-Bertalot and Metzeltin 1996 | Pl. 25: 10 |
| *Sellaphora saugerresii* (Desmazières) C.E. Wetzel et D.G. Mann | Wetzel et al. 2015 | P. 214: 112‒127 |
| * *Sellaphora stauroneioides* Lange-Bertalot | Lange-Bertalot and Metzeltin 1996 | Pl. 109: 24 |
| ** Sellaphora wallacei* (Reimer) Potapova et Ponader | Potapova and Ponader 2008 | P. 173, fig. 1C |
| *Sellaphora* sp. 1 |  |  |
| *Sellaphora* sp. 2 |  |  |
| *Sellaphora* sp. 3 |  |  |
| *Simonsenia* cf. *delognei* (Grunow) Lange-Bertalot | Hofmann et al. 2013 | Pl. 117: 47 |
| * *Stauroneis reichardtii* Lange-Bertalot, Cavacini, Tagliaventi et Alfinito | Bahls 2010 | P. 127, fig. 130401 |
| *Stauroneis* cf. *acidoclinatopsis* Van de Vijver and Lange-Bertalot | Bahls 2010 | P. 29, fig. 452601 (1) |
| *Stauroneis* cf. *schmidiae* R. Jahn et N. Abarca | Zimmermann et al. 2014 | Fig. 4.4: h |
| *Stauronei*s sp. 1 |  |  |
| *Stauroneis* sp. 2 |  |  |
| ** Stenopterobia delicatissima* (Lewis) Van Heurck | Krammer and Lange-Bertalot 1997b | Pl. 174: 6 and 10 |
| *Surirella angusta* Kützing | Metzeltin et al. 2005 | Pl. 221: 1‒7 |
| ** Surirella* *apiculata* var. *panduriformis* Frenguelli | Metzeltin et al. 2005 | Pl. 222: 7‒9 |
| *Surirella ovalis* Brébisson | Metzeltin et al. 2005 | Pl. 220: 1‒2 |
| *Surirella* cf. *pseudolinearis* Krasske | Metzeltin et al. 2005 | Pl. 225: 9 |
| *Surirella* sp. 1 |  |  |
| *Tryblionella apiculata* W. Gregory | Krammer and Lange-Bertalot 1997b | Pl. 35: 5 |
| *Tryblionella calida* (Grunow) D.G. Mann | Metzeltin et al. 2005 | Pl. 194: 5‒6 |
| *Tryblionella hungarica* (Grunow) D.G. Mann | Metzeltin et al. 2005 | Pl. 194: 10‒12 |
| *Ulnaria acus* (Kützing) Aboal | Hofmann et al. 2013 | Pl. 5: 3 |
| *Ulnaria ulna* (Nitzsch) Compère | Hofmann et al. 2013 | Pl. 5: 9 |

Bahls L (2010) *Stauroneis* in the Northern Rockies: 50 species of *Stauroneis* sensu stricto from western Montana, northern Idaho, northeastern Washington and southwestern Alberta, including 16 species described as new. Northwest Diatoms 4: 1‒172.

Håkansson H (2002) A compilation and evaluation of species in the general *Stephanodiscus*, *Cyclostephanos* and *Cyclotella* with a new genus in the family Stephanodiscaceae. Diatom Research 17(1): 1‒139. doi: 10.1080/0269249X.2002.9705534

Hamilton P (2010) *Brachysira microcephala*. Diatoms of the United States. October 31, 2016, from http://westerndiatoms.colorado.edu/taxa/species/brachysira_microcephala [accessed 31.10.2016]

Hofmann G, Lange‒Bertalot H, Werum M (2013) Diatomeen im Süßwasser–Benthos von Mitteleuropa. Bestimmungsflora Kieselalgen für die ökologische Praxis. Über 700 der häufigsten Arten und ihre Ökologie. Koeltz Scientific Books, Königstein, 1‒908.

Jahn R, Kusber W‒H, Romero OE (2009) *Cocconeis pediculus* Ehrenberg and *C. placentula* Ehrenberg var. *placentula* (Bacillariophyta): Typification and taxonomy. Fottea 9(2): 275‒288. doi: 10.5507/fot.2009.027

Jahn R, Abarca N, Gemeinholzer B, Mora D, Skibbe O, Kulikovskiy M, Gusev E, Kusber W‒H, Zimmermann J (2017) *Planothidium lanceolatum* and *Planothidium frequentissimum* reinvestigated with molecular methods and morphology: four new species and the taxonomic importance of the sinus and cavum. Diatom Research 32(1) 75‒107. doi: 10.1080/0269249X.2017.1312548

Kociolek P (2011a) *Nitzschia balcanica*. In Diatoms of the United States. http://westerndiatoms.colorado.edu/taxa/species/nitzschia_balcanica [accessed 01.09.2017]

Kociolek P (2011b) *Nitzschia palea*. In Diatoms of the United States. http://westerndiatoms.colorado.edu/taxa/species/nitzschia_palea_var._debilis [accessed 24.10.2016]

Kociolek P (2011c) *Nitzschia palea*. In Diatoms of the United States. http://westerndiatoms.colorado.edu/taxa/species/nitzschia_palea_var._tenuirostris [accessed 24.10.2016]

Kociolek JP, Laslandes B, Bennett D, Thomas E, Brady M, Graeff C (2014) Diatoms of the United States 1: taxonomy, ultrastructure and descriptions of new species and other rarely reported taxa from lake sediments in the western U.S.A. Bibliotheca Diatomologica 61. J. Cramer, Stuttgart, 1‒188.

Krammer K (1992) *Pinnularia*, eine Monographie der europäischen Taxa. Bibliotheca Diatomologica 26. J. Cramer, Berlin‒Stuttgart, 1‒353.

Krammer K (1997a) Die cymbelloiden Diatomeen. Eine Monographie del weltweit bekannten Taxa. Teil 1. Allgemeines und *Encyonema* Part. Bibliotheca Diatomologica 36. J. Cramer, Berlin‒Stuttgart, 1‒382.

Krammer K (1997b) Die cymbelloiden Diatomeen. Eine Monographie del weltweit bekannten Taxa. Teil 2. *Encyonema* part., *Encyonopsis* and *Cymbellopsis*. ‒ Bibliotheca Diatomologica 37. J. Cramer, Berlin‒Stuttgart, 1‒469.

Krammer K (2000) Diatoms of the European inland waters and comparable habitats. The genus *Pinnularia*. Diatoms of Europe 1. A.R.G. Gantner Verlag K.G., Ruggell. 1‒703.

Krammer K (2002) Diatoms of the European inland waters and comparable habitats. *Cymbella*. Diatoms of Europe 3. A.R.G. Gantner Verlag K.G., Ruggell, 1‒584.

Krammer K, Lange‒Bertalot H (1997a) Bacillariophyceae. 2. Teil: Naviculaceae. In: Ettl, H, Gerloff J, Heynig H, Mollenhauer D (Eds) Süßwasserflora von Mitteleuropa. 2/1. Gustav Fischer, Jena, 1‒876.

Krammer K, Lange‒Bertalot H (1997b) Bacillariophyceae. 2. Teil: Bacillariaceae, Epithemiaceae, Surirellaceae. In: Ettl H, Gerloff J, Heynig H, Mollenhauer D (Eds) Süßwasserflora von Mitteleuropa. 2/2. Gustav Fischer, Jena. 1‒610.

Krammer K, Lange‒Bertalot H (1991a) Bacillariophyceae. 3. Teil: Centrales, Fragilariaceae, Eunotiaceae. In: Ettl H, Gerloff J, Heynig H, Mollenhauer D (Eds) Süßwasserflora von Mitteleuropa. 2/3. Spektrum Akademischer Verlag, Heidelberg‒Berlin. 1‒598.

Krammer K, Lange‒Bertalot H (1991b) Bacillariophyceae. 4. Teil: Achnanthaceae. Kritische Ergänzungen zu *Navicula* (Lineolatae) und *Gomphonema* Gesamtliteraturverzeichnis Teil 1‒4. In: Ettl H, Gärtner G, Gerloff J, Heynig H, Mollenhauer D (Eds) Süßwasserflora von Mitteleuropa. 2/4. Gustav Fischer Verlag, Stuttgart‒Jena. 1‒4347.

Lange‒Bertalot H (1993) 85 Neue Taxa und über 100 weitere neu definierte Taxa ergänzend zur Süβwasserflora von Mitteleuropa Vol. 2/1‒4. Bibliotheca Diatomologica 27. J. Cramer, Berlin‒Stuttgart, 1‒ 454.

Lange‒Bertalot H (2001) Diatoms of the European inland waters and comparable habitats. *Navicula* sensu stricto. 10 genera separated from *Navicula* sensu lato. *Frustulia*. Diatoms of Europe 2. A.R.G. Gantner Verlag K.G., Ruggell, 1‒526.

Lange‒Bertalot H, Metzeltin D (1996) Indicators of oligotrophy: 800 taxa representative of three ecologically distinct lake types: carbonate buffered ‒ oligodystrophic ‒ weakly buffered soft water. Iconographia Diatomologica 2. Koeltz Scientific Books, Königstein, 1‒390.

Lange‒Bertalot H, Moser G (1994) *Brachysira*. Monographie der Gattung. Wichtige Indikator‒Species für das Gewässer Monitoring und *Naviculadicta* nov. gen. Ein Lösungsvorschlag zu dem Problem *Navicula* sensu lato ohne *Navicula* sensu stricto. Bibliotheca Diatomologica 29. J. Cramer, Berlin‒Stuttgart, 1–212.

Lange‒Bertalot H, Cavacini P, Tagliaventi N, Alfinito S (2003) Diatoms of Sardinia. Rare and 76 new species in rock pools and other ephemeral waters. Iconographia Diatomologica 12. A.R.G. Gantner Verlag K.G., Ruggell, 1‒438.

Lange‒Bertalot H, Bąk M, Witkowski A, Tagliaventi N (2011) Diatoms of the European inland waters and comparable habitats. *Eunotia* and some related genera. Diatoms of Europe 6. A.R.G. Gantner Verlag K.G., Ruggell, 1‒747.

Levkov Z (2009) Diatoms of the European inland waters and comparable habitats. *Amphora* sensu lato. Diatoms of Europe 5. A.R.G. Gantner Verlag K.G., Ruggell, : 1‒916.

Levkov Z, Krstic S, Metzeltin D, Nakov T (2007) Diatoms of lakes Prespa and Ohrid. About 500 taxa from ancient lake system. Iconographia Diatomologica 16. A.R.G. Gantner Verlag K.G., Ruggell, 1‒613.

Levkov Z, Metzeltin D, Pavlov A (2013) Diatoms of the European inland waters and comparable habitats. *Luticola* and *Luticolopsis*. Diatoms of Europe 7. Koeltz Scientific Books, Königstein, 1‒698.

Levkov Z, Mitić‒Kopanja D, Reichardt E (2016) Diatoms of the European inland waters and comparable habitats. The diatom genus *Gomphonema* in the Republic of Macedonia. Diatoms of Europe 8. Koeltz Botanical Books, Oberreifenberg, 1‒552.

Metzeltin D, Lange‒Bertalot H (1998) Tropical diatoms of South America I. About 700 predominantly rarely known or new taxa representative of the neotropical flora. Iconographia Diatomologica 5. Koeltz Scientific Books, Königstein, 1‒695.

Metzeltin D, Lange‒Bertalot H (2007) Tropical diatoms of South America II. Special remarks on biogeographic disjunction. Iconographia Diatomologica 18. A.R.G. Gantner Verlag K.G., Ruggell, 1‒877.

Metzeltin D, Lange‒Bertalot H, García‒Rodríguez F (2005) Diatoms of Uruguay. Compared with other taxa from South America and elsewhere. Iconographia Diatomologica 15. A.R.G. Gantner Verlag K.G., Ruggell, 1‒736.

Metzeltin D, Lange‒Bertalot H, Nergui S, Yanling L (2009) Diatoms in Mongolia. Iconographia Diatomologica 20. A.R.G. Gantner Verlag K.G., Ruggell, 1‒686.

Potapova MG, Ponader KC (2008) New species and combinations in the diatom genus *Sellaphora* (Sellaphoraceae) from southeastern United States. Harvard Papers in Botany 13(1): 171‒181. doi: 10.3100/1043-4534(2008)13[171:NSACIT]2.0.CO;2

Reichardt E (1999) Zur Revision der Gattung *Gomphonema*. Die Arten um *G. affine*/*insigne*, *G. angustatum*/*micropus*, *G. acuminatum* sowie gomphonemoide Diatomeen aus dem Oberoligozän in Böhmen. Iconographia Diatomologica 8. A.R.G. Gantner Verlag K.G., Ruggell. 1‒203.

Reichardt E (2015) *Gomphonema gracile* Ehrenberg sensu stricto et sensu auct. (Bacillariophyceae): A taxonomic revision. Nova Hedwigia 101(3/4): 367‒393. doi: 10.1127/nova_hedwigia/2015/0275

Rumrich U., Lange‒Bertalot H, Rumrich M (2000) Diatomeen der Anden. Von Venezuela bis Patagonien/Feuerland. Und zwei weitere Beiträge. Iconographia Diatomologica 9. A.R.G. Gantner Verlag K.G., Ruggell, 1‒673.

Stancheva R, Manoylov K, Gillett N (2009) Morphological variation of the *Caloneis schumanniana* species complex (Bacillariophyceae) from different environmental conditions in North American streams. Hydrobiologia 635(1): 157‒170. doi 10.1007/s10750-009-9908-4

Werum M, Lange‒Bertalot H, Reichardt E (2004) Diatoms in springs from Central Europe and elsewhere under the influence of hydrogeology and anthropogenic impacts: Diatomeen in Quellen unter hydrogeologischen und anthropogenen Einflüssen in Mitteleuropa und anderen Regionen. Iconographia Diatomologica 13. A.R.G. Gantner Verlag K.G., Ruggell, 1‒480.

Wetzel CE, Ector L (2015) Taxonomy and ecology of *Fragilaria microvaucheriae* sp. nov. and comparison with the type materials of *F. uliginosa* and *F. vaucheriae*. Cryptogamie, Algologie, 36(3): 271‒289. doi/10.7872/crya/v36.iss3.2015.271

Wetzel CE, Ector L, van de Vijver B, Compère P, Mann DG (2015) Morphology, typification and critical analysis of some ecologically important small naviculoid species (Bacillariophyta). Fottea 15(2): 203‒234. doi: 10.5507/fot.2015.020

Wetzel C E, van De Vijver B, Hoffmann L, Ector L (2013) *Planothidium incuriatum* sp. nov. a widely distributed datom species (Bacillaryophyta) and type analysis of *Planothidium biporomum*. Phytotaxa 138(1): 43‒57. doi: 10.11646/phytotaxa.138.1.6

Zimmermann J, Abarca N, Enk N, Skibbe O, Kusber W‒H, Jahn R (2014) Taxonomic reference libraries for environmental barcoding: a best practice example from diatom research. PloS one, 9(9), e108793. doi:10.1371/journal.pone.0108793
